# Integrated Network Pharmacology and Lipidomics to Reveal the Inhibitory Effect of Qingfei Oral Liquid on Excessive Autophagy in RSV-Induced Lung Inflammation

**DOI:** 10.3389/fphar.2021.777689

**Published:** 2021-12-01

**Authors:** Lili Lin, Li An, Hui Chen, Lu Feng, Mengjiang Lu, Yuling Liu, Chu Chu, Jinjun Shan, Tong Xie, Xiaorong Wang, Shouchuan Wang

**Affiliations:** ^1^ Jiangsu Key Laboratory of Pediatric Respiratory Disease, Institute of Pediatrics, Medical Metabolomics Center, Affiliated Hospital of Nanjing University of Chinese Medicine, Nanjing, China; ^2^ Key Laboratory of Acupuncture and Medicine Research of Ministry of Education, Nanjing University of Chinese Medicine, Nanjing, China; ^3^ Department of Pediatrics, Nanjing Pukou District Hospital of Traditional Chinese Medicine, Nanjing, China; ^4^ Department of Clinical Laboratory, Affiliated Hospital of Nanjing University of Chinese Medicine, Nanjing, China

**Keywords:** respiratory syncytial virus, qingfei oral liquid, network pharmacology, lipidomics, excessive autophagy, PI3K/AKT/mTOR, lipin-1

## Abstract

**Background:** Respiratory syncytial virus (RSV) can cause varying degrees of lung inflammation in children. Qingfei Oral Liquid (QF) is effective in treating childhood RSV-induced lung inflammation (RSV-LI) in clinics, but its pharmacological profiles and mechanisms remain unclear.

**Methods:** This study combined network Pharmacology, lipidomics, pharmacodynamics, and pathway validation to evaluate the therapeutic mechanisms of QF. Using Cytoscape (v3.8.2) and enrichment analyses from the Kyoto Encyclopedia of Genes and Genomes (KEGG) and Gene Ontology (GO), a global view of the putative compound-target-pathway network was created. The corresponding lipidomic profiles were then used to detect differently activated lipids, revealing the metabolic pathway, using ultra-high-performance liquid chromatography linked to hybrid Quadrupole-Exactive Orbitrap mass spectrometry (UHPLC-Q-Exactive Orbitrap MS). Meanwhile, the *in vivo* efficiency of QF, the enrichment pathway, and the excessive autophagy inhibition mechanisms were validated in RSV-infected mice models.

**Results:** The network pharmacology results demonstrated 117 active compounds acted directly upon 101 core targets of QF against RSV-LI. The most significantly enriched pathway was the PI3K/Akt/mTOR signaling pathway (*p* < 0.05). In addition, untargeted lipidomics were performed, and it was revealed that higher lung levels of DAG 30:0, DAG 30:5, DAG 32:0, DAG 16:0_18:0, DAG 17:0_17:0, DAG 34:1, DAG 36:0, DAG 36:1 in the RSV-LI group were decreased after QF administration (*FDR* < 0.05, FC > 1.2). Lipin-1, a key enzyme in DAG synthesis, was increased in the RSV-LI mouse model. Animal experiments further validated that QF inhibited the PI3K/Akt/mTOR signaling pathway, with lower lung levels of phosphorylated PI3K, AKT and mTOR, as well as its related proteins of lipin-1 and VPS34 (*p* < 0.01). Finally, pharmacodynamic investigations indicated that QF reduced airway inflammation caused by excessive autophagy by decreasing lung levels of RSV F and G proteins, Beclin-1, Atg5, and LC3B II, IL-1 and TNF-α (*p* < 0.05).

**Conclusion:** Lipidomic-based network pharmacology, along with experimental validation, may be effective approaches for illustrating the therapeutic mechanism of QF in the treatment of RSV-LI.

## Introduction

Respiratory syncytial virus (RSV) is the most common causative agent leading to respiratory tract infection worldwide (Borchers et al., 2013). According to extensive epidemiological analysis, immunocompromised populations are more vulnerable to developing bronchiolitis and pneumonia induced by RSV infection, accounting for the high rates of hospitalizations and deaths among preterm babies and infants (Rey-Jurado and Kalergis, 2017; Seidenberg, 2019). Unlike mild symptoms, RSV- induced lung inflammation (RSV-LI) is closely associated with severe wheezing and hyperreactive airways, and asthma exacerbation at young ages (Tsukagoshi et al., 2013; Bianchini et al., 2020). Despite its remarkable threat to human health, population-based data on the practical and safe therapeutic options for treating this viral disease are lacking (Griffiths et al., 2017). To date, only palivizumab, a monoclonal antibody, has been applied to prevent severe illness caused by RSV infection at high cost (Perk and Özdil, 2018). Therefore, intense exploration should be focused on developing an effective and affordable strategy for the prevention and treatment of RSV-LI.

Traditional Chinese medicine (TCM), a unique and holistic theory system, has been employed for preventing and treating viral diseases for thousands of years (Oravecz and Mészáros, 2012). Qingfei Oral Liquid (QF) is derived from a classic prescription called “Maxin Shigan Decoction,” and composed of *Ephedra sinica Stapf.* (Ma Huang 5g), *Prunus armeniaca L.* (Ku Xingren 12g), *Gypsum Fibrosum* (Sheng Shigao 40g), *Morus alba L.* (Sang Baipi 12g), *Lepidium virginicum L.* (Ting Lizi 10g), *Angelica decursiva* (Zihua Qianhu 12g), *Bistorta officinalis Delarbre* (Quan Shen 15g), *Reynoutria japonica Houtt.* (Hu Zhang 15g), *Bombyx mori Linnaeus.* (Jiang Can 8g), and *Salvia miltiorrhiza Bunge* (Dan Shen 8g). Our previous research confirmed that QF can significantly relieve the symptoms and signs of fever, wheezing, and pulmonary rales in children with RSV pneumonia (Wang et al., 2016). *In vitro* experiments showed that QF prevented RSV proliferation by inhibiting virus membrane fusion with host cells (Yuan et al., 2009). Furthermore, it alleviated RSV-LI by reversing immune cell imbalance and decreasing inflammatory responses (Zhu et al., 2014; Zou et al., 2018). However, previous results only reflect the global effects of QF against RSV-LI, and targets for drug intervention have not been clarified.

Due to the multi-target and multi-component characteristics of TCM, it is difficult to identify the specific components and targets of TCM formulas (Li and Zhang, 2013). In recent years, bioinformatics in the form of network pharmacology has been widely applied to reveal interactions between targets and diseases through bioactive component identification, network construction, and pathway enrichment (Zhu et al., 2021). In addition, lipidomics, a system biology technique for large-scale determination of individual lipid species, has also been applied to investigate various human diseases. Given the systematic characteristics embodied in omics approaches, application of lipidomics can generate a complete atlas of the metabolic landscape, and enable comprehensive analysis to identify critical metabolic factors in disease pathology (Wang R. et al., 2020). Thus, systematic features of network pharmacology and lipidomic research can be combined for a comprehensive investigation of TCM formulas. By establishing an omics-based network, drug action can be unveiled through multi-layer information, thereby providing a promising alternative for accelerating rational drug design and better exploring the mechanism underlying TCM therapy (Caberlotto and Lauria, 2015). Despite several significant TCM achievements in blocking viral infection, the critical role of QF against RSV-LI has not been investigated using bioinformatics and omics analyses.

**FIGURE 1:**
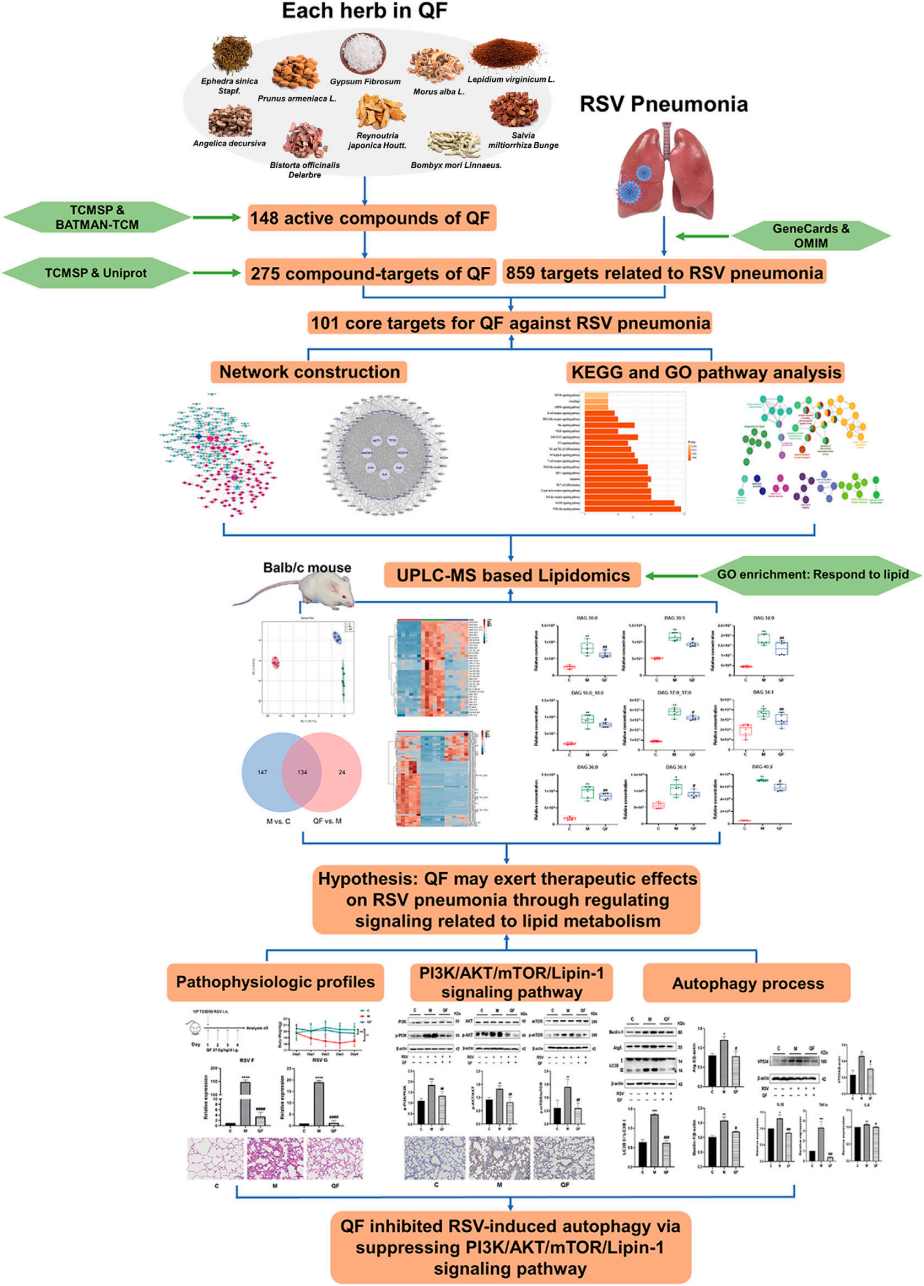
Study design. Abbreviations: QF, Qingfei oral liquid; RSV, respiratory syncytial virus.

Here, integrated network pharmacology with lipidomics, we identified and validated the active compounds, core targets, signaling pathways, and disturbed lipids involved in QF treatment of RSV-LI in BALB/c mice models (Figure 1).

## Methods and Materials

### Network Pharmacology Analysis

Chemical compounds of QF were obtained from the databases of traditional Chinese medicine systems pharmacology (TCMSP, http://tcmspw.com/tcmsp.php) and bioinformatics analysis tool for molecular mechanism of traditional Chinese medicine (BATMAN-TCM, http://bionet.ncpsb.org/batman-tcm) (Ru et al., 2014; Liu et al., 2016). Active compounds were screened with oral bioavailability (OB)≥30% and drug-likeness (DL)≥0.18. Protein targets of the active components in QF were converted to official symbols by checking with reviewed Homo sapiens gene list in the Protein Knowledgebase (UniProtKB, http://www.uniprot.org/) (Tao et al., 2020). The disease-related targets were collected by inputting keywords “respiratory syncytial virus pneumonia,” “RSV pneumonia” and “RSV-induced lung inflammation” into online platforms including GeneCards (http://www. genecards. org/) and online Mendelian inheritance in man (OMIM; http://www.omim.org/) (Stelzer et al., 2016; Amberger and Hamosh, 2017). The common target genes were selected out as QF targets in the treatment of RSV-LI. The core targets were further analyzed by STRING (https://string-db.org/cgi/input.pl) to construct Protein–Protein Interactions (PPI) network (Szklarczyk et al., 2017). The enrichment analysis in the Kyoto Encyclopedia of Genes and Genomes (KEGG) and Gene Ontology (GO) were performed to demonstrate the main biological functions and signal transductions involved by core targets. The top 20 KEGG pathways (*p* < 0.05) were displayed in a histogram using the MetaScape Bioinformatics Resource database (http://metascape.org/) (Zhou et al., 2019). GO enrichment was conducted by ClueGO, a plug-in of Cytoscape (v3.8.2) software (http://www.cytoscape.org/) to visualize biological process (BP), cellular component (CC) and molecular function (MF) enrichments of core targets.

### LC-MS/MS Analysis of QF

QF was made up of the following ingredients: *Ephedra sinica Stapf.* (batch No. 190807, 5g), *Prunus armeniaca L.* (batch No.191321, 12g), *Gypsum Fibrosum* (batch No. 191201, 40g), *Morus alba L.* (batch No. 190407, 12g), *Lepidium virginicum L.* (batch No. 190312, 10g), *Angelica decursiva* (batch No. 190129, 12g), *Bistorta officinalis Delarbre* (batch No. 191227, 15g), *Reynoutria japonica Houtt.* (batch No. 191115, 15g), *Bombyx mori Linnaeus.* (batch No. 191104, 8g) and *Salvia miltiorrhiza Bunge* (batch No. 191226, 8g). The ten products were purchased from Tongling Hetian Chinese Medicine herbal tablets Co., Ltd. Anhui, China. QF was prepared in accordance with standard operating procedures (Wang et al., 2003). In brief, the ten drugs were mixed and decocted twice in filtered water for 1 hour at 100°C, and the filtrate was concentrated to a final concentration of 3.65 g/ml using a rotary evaporator (YRE-2000A, China). The concentrating agent was kept at −20°C for future use. The fingerprinting analysis of QF (30 ug/ml) was performed in both negative and positive ionization modes using a Linear Ion Trap Quadrupole-Orbitrap Mass Spectrometer (LTQ-Obitrap MS; Thermo Fisher Scientific, USA).

### BALB/c Mouse Model of RSV-LI

Hep2 cells (human laryngeal carcinoma cells; China Center for Type Culture Collection, Wuhan, China) were cultured with DMEM medium (10% FBS, 100 U/ml penicillin, 0.1 mg/ml streptomycin) to provide condition for RSV strain A2 (China Center for Type Culture Collection, Wuhan, China). The viral supernatant was collected after 4–5 days and a plaque assay was performed to ensure that the virulence reached 1*10^6 PFU/ml. The viral solution was stored in liquid nitrogen after being centrifuged for 10 min at 3,000 rpm at 4°C. The animal experiment in this study was approved by the ethics committee of the Laboratory Animal Center at Nanjing University of Chinese Medicine (ethical # 201912A009). The *in vitro* and *in vivo* experiments were conducted out in Yangzhou University’s biosafety level-2 laboratory. A total of 18 female BALB/c mice (aged 6–8 weeks, 18 ± 2 g) were purchased from the Laboratory Animal Department of Shanghai Family Planning Research Institute (permission number: 202116007) and randomly assigned to three groups: a control group (C), an RSV group (M) and a QF group (*n* = 6 per group). The RSV and QF groups received 80 μl RSV suspension intranasally under inhalation anesthesia on the first day of modeling, whereas the control group received equal DMEM medium. QF (27.6 g/kg/day) was delivered intragastrically for four consecutive days, equivalent to the clinical human dosage as assessed by host weight (Zhu et al., 2021). On the fifth day after intervention, mice were sacrificed. The body weight, energy level, diet, drinking water, fur color, and activity of the mice in each group were recorded daily.

### Sample Collection and Histomorphology Assay

After the mice were euthanized, middle lobe of right lung was fixed in 4% neutral buffered paraformaldehyde for 12 h dehydration before being embedded in paraffin wax and stained with hematoxylin and eosin (H&E). Tissue lesions and inflammatory cell infiltrates were measured and captured under a light microscope at high magnification (200×) (OLYMPUS, Japan). Lung tissue was divided into 20 mg aliquots for lipidomic analysis and the remaining was stored at −80°C for the biochemical indexes including PI3K, p-PI3K, AKT, p-AKT, mTOR, p-mTOR, Lipin-1, Beclin-1, Atg5, LC3B, VPS34, IL-1β, IL-6, and TNF-α.

### Lipidomic Analysis

#### UPLC- Q-Exactive/MS Measurement

To perform a comprehensive lipidomic profiling, the sample preparation strategy for lung tissue based on liquid-liquid MTBE extraction was previously established (Du et al., 2015). Briefly, 20 μl lung tissues homogenate was transferred into a clean tube and mixed with 225 μl cold methanol solution containing 10 μg/ml internal standards, including lyso PE (17:1) (batch number: LM171LPE-11), LPC17:0 (batch number: LM170LPC-30), PE (17:0/17:0) (batch number: LM170PE-19) purchased from Avanti Polar Lipids Company. After vortexing for 10 s, 750 μl Methyl tert-butyl ether (MTBE, ROE, USA) was added, and the mixture were shaken for 10 min at 4°C. The samples were vortexed for 10 s after adding 188 μl of deionized water, and then centrifuged at 18,000 rpm at 4°C in an Integrated SpeedVac apparatus (Thermo Fisher Scientific, USA). The upper (organic) phase, primarily lipids, was transferred to new tubes and dried in a Savant SPD1010 vacuum centrifuge concentrator (Thermo Fisher Scientific, USA). Finally, for LC-MS analysis, the upper phase lipids were reconstituted in 110 μl methanol: toluene (9:1) solution. The preparation was carried out by combining equal aliquots of 10 μl from each sample, and they were pretreated in the same way as the samples. Qulity control (QC) pool samples were injected before the samples, and one QC injection was placed per ten samples on a regular basis.

Untargeted lipidomics was conducted with a Dionex UltiMate 3000 ultra-high-performance liquid chromatography (UHPLC, Santa Clara, USA) coupled to Q Exactive Orbitrap Mass Spectrometers (Q-Exactive-MS; Thermo Fisher Scientific, USA). For gradient elution, a 2 μl sample solution was injected onto a Waters ACQUITY UPLC CSH C18 (100 mm, 2.1 mm, 1.7 mm) kept at 65°C. Mobile phase A was 40% acetonitrile in water, whereas mobile phase B was a 9:1 mixture of isopropanol and acetonitrile containing 5 mM ammonium formate and 0.1% formic acid. The specific elution gradient was as follows: 0–2 min, 15–30% B; 2–2.5 min, 30–48% B; 2.5–11 min, 48–82% B; 11–11.5 min, 82–99% B; 11.5–12 min, 99% B; 12–13 min, 99–15% B; 13–15 min, 15% B. The flow rate was 0.6 ml/min. The MS experiment was carried out in positive ion mode, with a heated electrospray ionization source (3.5 kV spray voltage). In full-scan mode, the mass analyzer had a resolving power of 60000 FWHM and a scan range of 150–1,500 m/z.

### Data Processing and Statistical Analysis

ABF converter (http://www.reifycs.com/AbfConverter) was used to convert raw data from the Xcalibur 2.2 program (Thermo Fisher Scientific, USA) to ABF formats. The files were then analyzed using the MS-DIAL (v4.10) software tool. The parameters of MS-DIAL were described in the previous study (Yang et al., 2014). For lipid identification, the public LipidBlast library was used with accurate mass and MS/MS matching. Adduct ions were set as + H, +NH4 and +Na for the positive ion mode. MetaboAnalyst 5.0 (http://www.metaboanalyst.ca) was used to further normalize multidimensional data following median and pareto scaling. The difference in lipidomic profiles between the control, model, and QF groups was represented in the principal component analysis (PCA) model. False discovery rate (FDR <0.05, Kruskal-Wallis test) and fold change (FC > 1.2 or <0.833, ratio of median) were used to screen for significant lipids. The number of screened lipids in each of the two groups was represented by a Venn diagram. Furthermore, heatmaps revealed the pattern of key lipids that were expressed in opposite directions between the two groups. In the boxplots, the FDR values were used to compare the relative concentrations of diacylglycerol (DAG).

### Biochemical Analysis

#### Quantitative Real-Time PCR

Total RNA extractions were performed with a FastPure Tissue Total RNA Isolation Kit (Vazyme, China) in accordance with the manufacturer’s instructions. Total RNA concentration and purity were determined using a Bio Photometer (Eppendorf, Germany). Extracted RNA was reverse-transcribed into cDNA using a Reverse transcription and cDNA synthesis kit (Abm, China). Quantitative real-time PCR was conducted on a 384-well plate under QuantStudio 7 Flex Real-Time PCR System (Thermo Fisher Scientific, USA). Data was normalized and processed with 2^−ΔΔCT^ method by taking the housekeeping gene GAPDH as reference standard.

### Western Blotting

Protein was extracted from lung tissues on ice for 30 min using RIPA lysis buffer with proteinase and phosphatase inhibitors (100:1:1). The tissue lysate was then centrifuged for 30 min at 13,000 rpm and 4°C. Using bovine serum albumin as a reference, protein content was determined using a BCA Protein Assay Kit (Thermo Fisher Scientific, USA). 30 μg total proteins of lung tissue lysate were run on 10% tris-glycine SDS-PAGE gel (Biosharp), 80 V for 0.5 h and 110 V for 1 h. Gels were transferred to a polyvinylidene difluoride (PVDF) membrane by Trans-Blot (Bio-Rad, USA), and blocked with 5% BSA for 1 h. The bands were incubated overnight at 4°C with primary antibodies: PI3K (1:1,000, CST, USA), p-PI3K (1:1,000, CST, USA), AKT (1:1,000, CST, USA), p-AKT (1:2000, CST, USA), mTOR (1:1,000, CST, USA), p-mTOR (1:1,000, CST, USA), Beclin-1 (1:1,000, CST, USA), Atg5 (1:1,000, CST, USA), LC3B (1:1,000, CST, USA), VPS34 (1:500, Proteintech, China), β-actin (1:20000, Proteintech, China). Proteins on the band were observed using a ChemiDoc XRS Gel Imaging System (Bio-Rad, USA) after 1 h at room temperature incubation with the appropriate secondary antibodies (Fcmacs, China). ImageJ software was used to examine the protein expression of each group (v1.8.0).

### Immunohistochemistry Assay

Lung tissue paraffin slides were deparaffinized in xylene, rehydrated using an ethanol gradient, antigen-retrieved in a 10 mM sodium citrate buffer (pH 6), blocked with 3% H2O2, and permeabilized with 1% BSA. The sections were then blocked with Lipin-1 antibody (1:500, Abcam, USA) overnight at 4°C. Afterwards, the secondary antibody (1:250, MaxVision, China) was added and incubated at room temperature for 20 min. After DAB staining, the slices were stained with hematoxylin and eosin for 10 min, dehydrated, and sealed in neutral resin. Lipin-1 protein expression levels were observed in different groups using a light microscope at high magnification (200×).

### Statistical Analysis

GraphPad Prism software (v8.0.2) was used to analyze and visualize all of the biochemical data. The standard deviation is shown by standard error of the mean (SEM). One-way analysis of variance (ANOVA) with the Student-Newman-Keuls post hoc test was used for multi-group comparisons, with *p* < 0.05 considered significant.

## Results

### Quality Control of QF by UHPLC-ESI/LTQ-Orbitrap-MS

Detailed information on formula composition of QF was shown in Table 1. The chemical profile of QF was originally investigated using UPLC-ESI/LTQ-Orbitrap-MS. Based on the reference compounds, a total of six components from 10 crude materials were identified qualitatively. The typical total ion chromatograms (TICs) are shown in Supplementary Figure S1. QF’s primary bioactive components, including Catechin (C15H14O6), Luteolin (C15H10O6), Quercetin (C15H10O7), Naringenin (C15H12O5), Kaempferol (C15H10O6), and Tanshinone IIA (C19H18O3), were processed under quality control (Supplementary Table S1). The detail parameters of the quality control were listed in supplementary file.

**TABLE 1:** Composition of QF.

Latin name	Family	Part use	Weight use (g)	Chinese name
*Ephedra sinica Stapf*	Ephedraceae	Stem	5	Ma Huang
*Prunus armeniaca L.*	Rosacea	Seed	12	Xingren
*Gypsum Fibrosum*	Anhydrite	Mineral	40	Sheng Shigao
*Morus alba L.*	Moraceae	Velamen	12	Sang Baipi
Lepidium virginicum L.	Brassicaceae	Seed	10	Ting Lizi
*Angelica decursiva*	Umbelliferae	Root	12	Zihua Qianhu
*Bistorta officinalis Delarbre*	Polygonaceae	Rhizome	15	Quan Shen
*Reynoutria japonica Houtt.*	Polygonaceae	Rhizome, Root	15	Hu Zhang
*Bombyx mori Linnaeus.*	Saturniidae	Whole body	8	Jiang Can
*Salvia miltiorrhiza Bunge*	Labiatae	Rhizome, Root	8	Dan Shen

Abbreviations: QF Qingfei oral liquid.

### Curative Effects of QF Against RSV-LI

**FIGURE 2:**
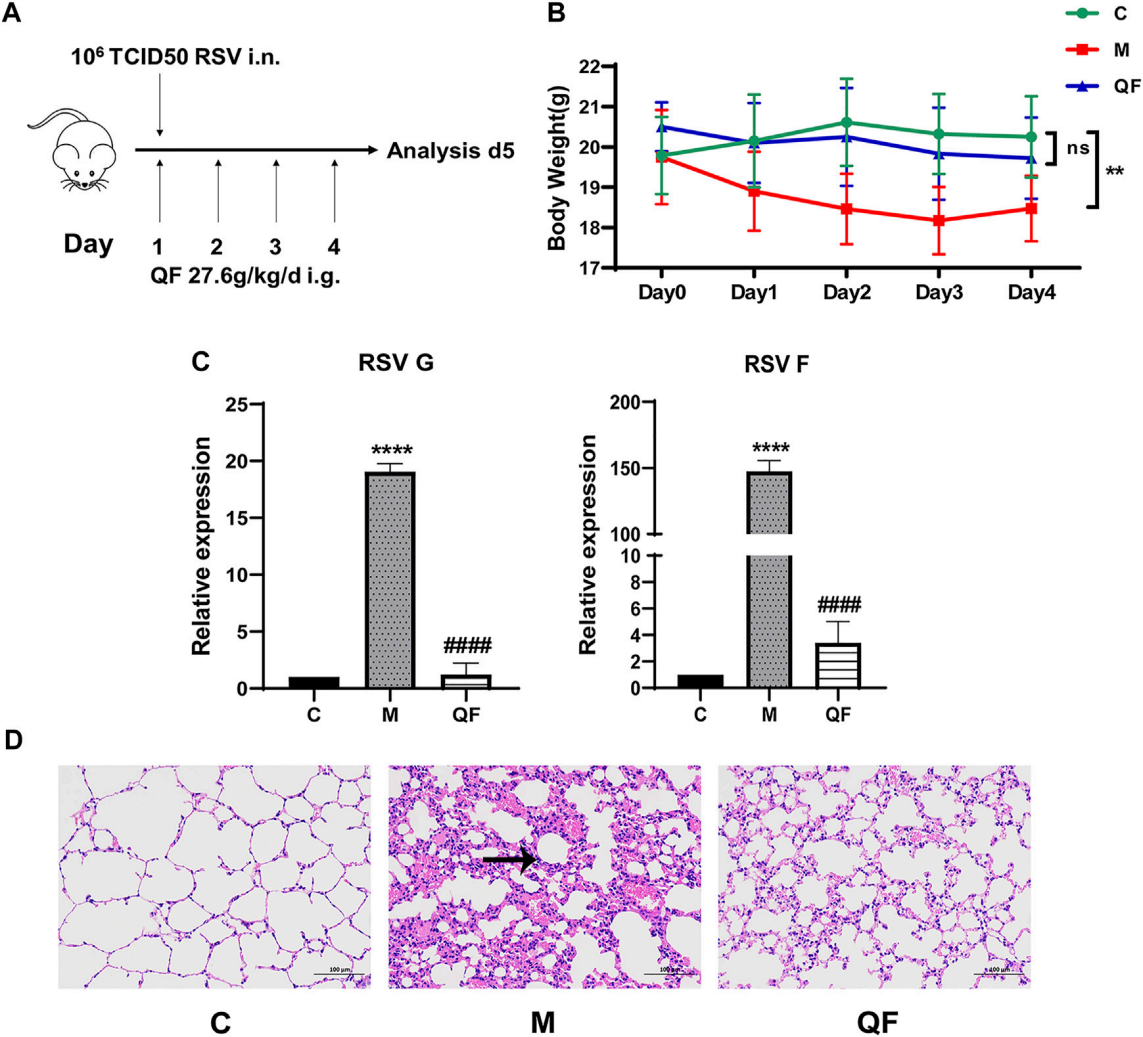
Evaluation of RSV-induced lung inflammation mouse model and efficacy of QF. **(A)** Schematic diagram of animal modeling and administration. **(B)** Weight changes of mice in control **(C)**, model (M), and therapy (QF) groups from day 0 to day 4. Values are expressed as mean ± standard error of the mean (SEM; n = 6, * compared with control group, **p < 0.01, ns = not significant). **(C)** Transcription levels of RSV F and RSV G proteins in lung tissues of mice in control (C), model (M), and therapy (QF) groups. Values are expressed as mean ± SEM (n = 6, * compared with control group, # compared with model group, ****p < 0.0001, ^####^ *p* < 0.0001. **(D)** H&E staining to evaluate pulmonary histopathological damage in control (C), model (M), and therapy (QF) groups (200× magnification). The black arrow indicated the evident inflammatory cell infiltration in lung tissue. Abbreviations: QF, Qingfei oral liquid; RSV, respiratory syncytial virus.

To develop a BALB/c mouse model of RSV-LI, 80 μl RSV suspension (1*10^6^ PFU/ml) intranasally under inhalation anesthesia on the first day of modeling (Figure 2A). Body weight (BW) changes revealed that RSV infection led to a significant weight loss within 24 h of nasal drip, with the mean value reaching the lowest level on the third day of modeling compared to controls (*p<0.01). Such weight loss, however, was successfully avoided in mice given QF therapy (*p>0.05 versus control, Figure 2B). The expression of RSV F and G proteins in lung tissues represented viral load. These two indicators’ transcription levels were evidently increased in the model group (*p<0.0001, versus control, Figure 2C), but they were significantly reduced in the QF group (^#^ *p*<0.0001, versus model; Figure 2C). Figure 2D showed HE staining of pulmonary pathologies in the three groups. In RSV-LI mice model, there was clear evidence of alveolar wall damage and inflammatory cell infiltration surrounding the bronchi, which was significantly alleviated after QF treatment.

### Identification of Compound-Targets-Pathway Network for QF Against RSV-LI

**FIGURE 3:**
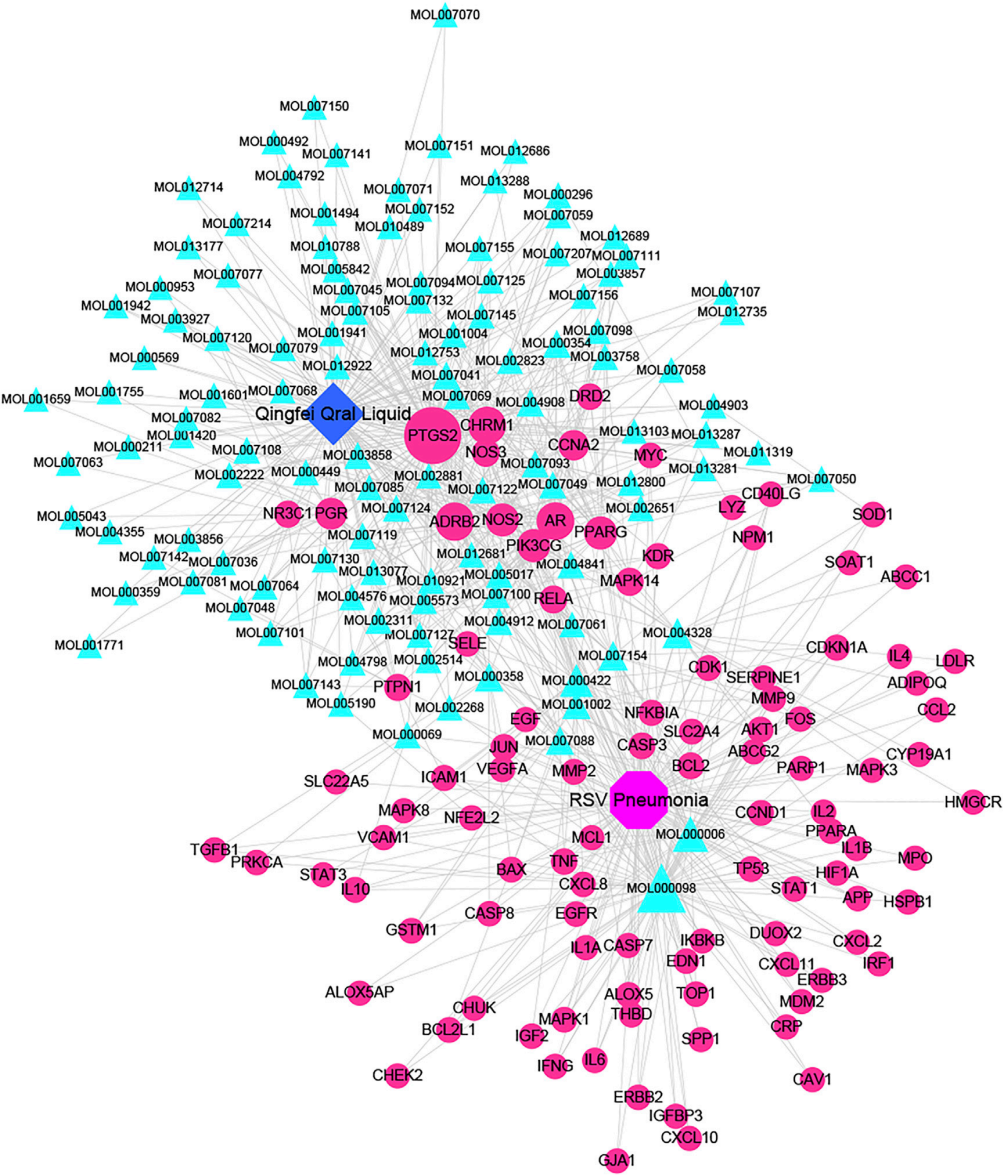
Active compound-core target network of QF in the treatment of RSV-induced lung inflammation. Blue triangle nodes represent the molecular identification (MOL ID) number of active components in QF, and red circle nodes represent the core targets. Edges represent interactions between compounds and targets. The size of each node is positively correlated with the degree value. Abbreviations: QF, Qingfei oral liquid; RSV, respiratory syncytial virus.

A total of 148 active compounds were identified in QF after integrating information from databases in TCMSP and BATMAN-TCM Specifically, 23 of them were found in *Ephedra sinica Stapf* (Ma Huang), 19 in *Prunus armeniaca L.* (Xingren), 12 in *Lepidium virginicum L.* (Ting Lizi), 27 in *Morus alba L.* (Sang Baipi), 8 in *Angelica decursiva* (Zihua Qianhu), 5 in *Bistorta officinalis Delarbre* (Quan Shen), 10 in *Reynoutria japonica Houtt.* (Hu Zhang), 1 in *Bombyx mori Linnaeus.* (Jiang Can), and 69 in *Salvia miltiorrhiza Bunge* (Dan Shen). Quercetin (MOL000098), Luteolin (MOL000006), Kaempferol (MOL000422), Naringenin (MOL004328), Tanshinone IIA (MOL007154), Beta-sitosterol (MOL000358), Cryptotanshinone (MOL007088), Ellagic acid (MOL001002), Isorhamnetin (MOL000354) and Dihydrotanshinlactone (MOL007100) were the top ten active chemicals for QF in the treatment of RSV-LI (Table 2). By referring to target mappings in TCMSP and translating protein names to official symbols on the UniProt database, 275 compound-related targets were identified. Furthermore, 859 disease-related targets were discovered in the GeneCards and OMIM databases. Finally, 101 symbols were identified as important targets for QF against RSV-LI by evaluating the shared parts in the two segments (Table 3). The network in Figure 3 constructed with 101 main targets and their matching 117 active molecules demonstrated the unique therapeutic benefits of QF working on RSV-LI. Based on interactions, active components primarily worked on targets such as Prostaglandin G/H synthase 2 (PGST2), Beta-2 adrenergic receptor (ADRB2), Muscarinic acetylcholine receptor M1 (CHRM1), Androgen receptor (AR), Nitric oxide synthase (NOS2), Peroxisome proliferator-activated receptor gamma (PPARG), and Phosphatidylinositol 4,5-bisphosphate 3-kinase catalytic subunit gamma isoform (PIK3CG).

**TABLE 2:** Information of the top 10 active compounds for QF in the treatment of RSV pneumonia.

MOL ID	Active ingredient	Molecular structure	Molecular weight	OB%	DL	Degree
MOL000098	Quercetin	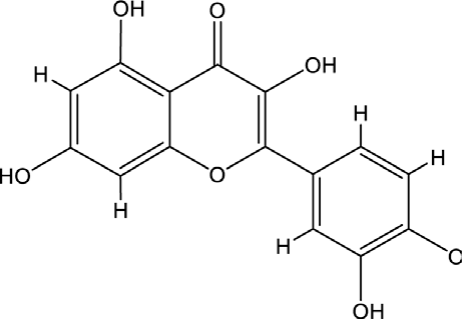	302.25	46.43	0.28	75
MOL000006	Luteolin	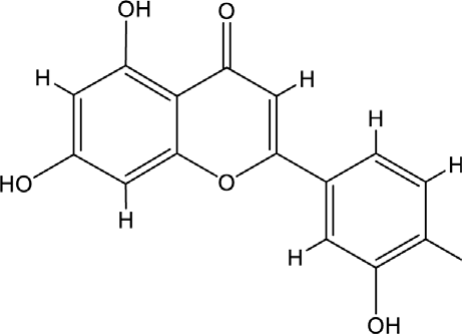	286.25	36.16	0.25	34
MOL000422	Kaempferol	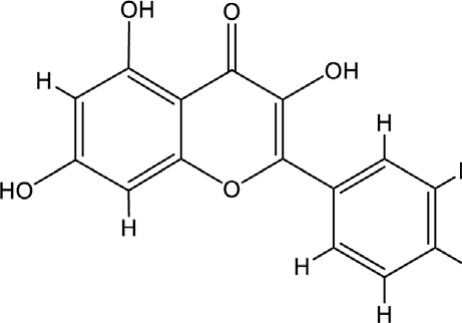	286.25	41.88	0.24	26
MOL004328	Naringenin	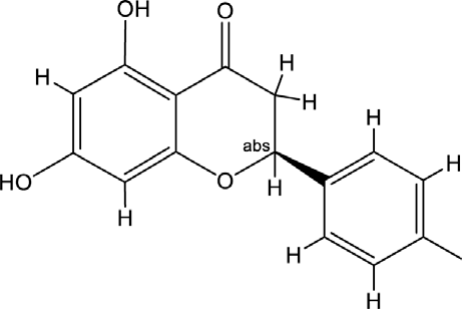	272.27	59.29	0.21	18
MOL007154	Tanshinone IIA	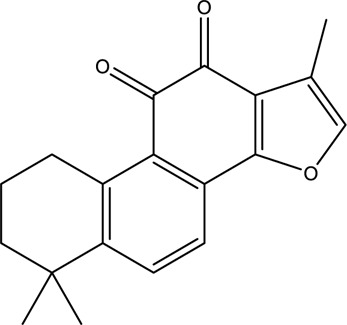	294.37	49.89	0.40	16
MOL000358	Beta-sitosterol	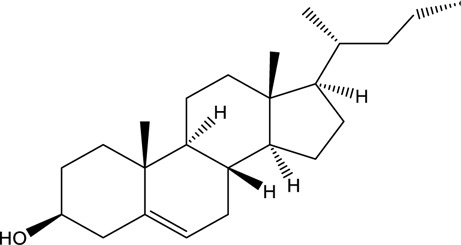	414.79	36.91	0.75	13
MOL007088	Cryptotanshinone	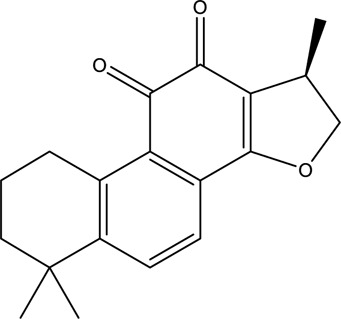	296.4	52.34	0.40	12
MOL001002	Ellagic acid	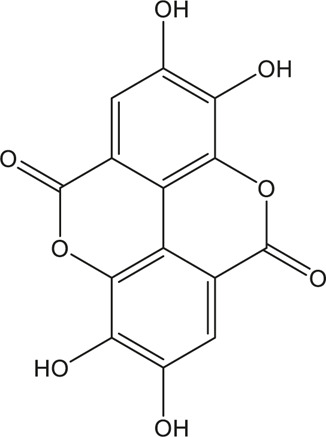	302.2	43.06	0.43	12
MOL000354	Isorhamnetin	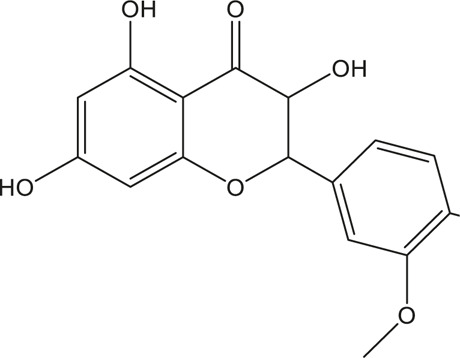	316.28	49.6	0.31	11
MOL007100	Dihydrotanshinlactone	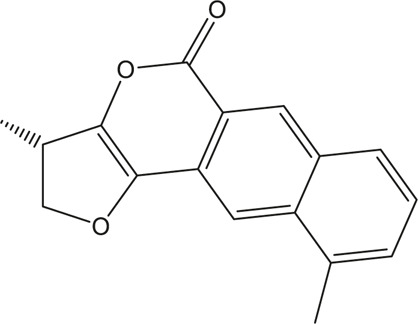	266.31	38.68	0.32	10

Abbreviations: QF Qingfei oral liquid; RSV respiratory syncytial virus; OB oral bioavailability; DL drug likeness.

**TABLE 3:** Information of core targets for QF in the treatment of RSV pneumonia.

No	Gene name	Protein name	Degree
1	AKT1	RAC-alpha serine/threonine-protein kinase	89
2	TP53	Cellular tumor antigen p53	83
3	MAPK3	Mitogen-activated protein kinase 3	82
4	VEGFA	Vascular endothelial growth factor A	82
5	TNF	Tumor necrosis factor	81
6	IL6	Interleukin-6	81
7	JUN	Transcription factor AP-1	81
8	CASP3	Caspase-3	78
9	MAPK8	Mitogen-activated protein kinase 8	74
10	STAT3	Signal transducer and activator of transcription 3	74
11	PTGS2	Prostaglandin G/H synthase 2	72
12	MAPK1	Mitogen-activated protein kinase 1	71
13	EGF	Pro-epidermal growth factor	71
14	EGFR	Epidermal growth factor receptor	71
15	MYC	Myc proto-oncogene protein	70
16	MMP9	Matrix metalloproteinase-9	69
17	CXCL8	Interleukin-8	68
18	IL1B	Interleukin-1 beta	67
19	CCL2	C-C motif chemokine 2	65
20	IL10	Interleukin-10	64
21	CCND1	G1/S-specific cyclin-D1	63
22	MAPK14	Mitogen-activated protein kinase 14	61
23	FOS	Proto-oncogene c-Fos	61
24	RELA	Transcription factor p65	59
25	PPARG	Peroxisome proliferator-activated receptor gamma	59
26	ICAM1	Intercellular adhesion molecule 1	58
27	MMP2	72 kDa type IV collagenase	57
28	ERBB2	Receptor tyrosine-protein kinase erbB-2	57
29	IL4	Interleukin-4	56
30	BCL2L1	Bcl-2-like protein 1	55
31	CASP8	Caspase-8	53
32	NOS3	Nitric oxide synthase, endothelial	53
33	IFNG	Interferon gamma	52
34	STAT1	Signal transducer and activator of transcription 1-alpha/beta	51
35	EDN1	Endothelin-1	51
36	IL2	Interleukin-2	51
37	SERPINE1	Plasminogen activator inhibitor 1	51
38	VCAM1	Vascular cell adhesion protein 1	50
39	KDR	Vascular endothelial growth factor receptor 2	50
40	SPP1	Osteopontin	48
41	AR	Androgen receptor	48
42	TGFB1	Transforming growth factor beta-1 proprotein	47
43	NFKBIA	NF-kappa-B inhibitor alpha	47
44	HIF1A	Hypoxia-inducible factor 1-alpha	46
45	CDKN1A	Cyclin-dependent kinase inhibitor 1	45
46	NOS2	Nitric oxide synthase, inducible	45
47	APP	Amyloid-beta precursor protein	44
48	MPO	Myeloperoxidase	44
49	MCL1	Induced myeloid leukemia cell differentiation protein Mcl-1	43
50	NR3C1	Glucocorticoid receptor	43
51	CAV1	Caveolin-1	43
52	MDM2	E3 ubiquitin-protein ligase Mdm2	43
53	CRP	C-reactive protein	42
54	CD40LG	CD40 ligand	41
55	CXCL10	C-X-C motif chemokine 10	41
56	ADIPOQ	Adiponectin	41
57	SELE	E-selectin	38
58	PGR	Progesterone receptor	36
59	PARP1	Poly [ADP-ribose] polymerase 1	35
60	IRF1	Interferon regulatory factor 1	35
61	CXCL2	C-X-C motif chemokine 2	34
62	IGF2	Insulin-like growth factor II	34
63	IL1A	Interleukin-1 alpha	32
64	SOD1	Superoxide dismutase [Cu-Zn]	32
65	HSPB1	Heat shock protein beta-1	31
66	NFE2L2	Nuclear factor erythroid 2-related factor 2	31
67	IGFBP3	Insulin-like growth factor-binding protein 3	30
68	CCNA2	Cyclin-A2	29
69	GJA1	Gap junction alpha-1 protein	29
70	IKBKB	Inhibitor of nuclear factor kappa-B kinase subunit beta	28
71	CASP7	Caspase-7	27
72	CDK1	Cyclin-dependent kinase 1	27
73	PTPN1	Tyrosine-protein phosphatase non-receptor type 1	27
74	LDLR	Low-density lipoprotein receptor	26
75	PPARA	Peroxisome proliferator-activated receptor alpha	26
76	ABCG2	Broad substrate specificity ATP-binding cassette transporter ABCG2	25
77	ALOX5	Polyunsaturated fatty acid 5-lipoxygenase	24
78	CXCL11	C-X-C motif chemokine 11	23
79	CYP19A1	Aromatase	23
80	SLC2A4	Solute carrier family 2, facilitated glucose transporter member 4	23
81	BCL2	Apoptosis regulator Bcl-2	22
82	PRKCA	Protein kinase C alpha type	22
83	CHEK2	Serine/threonine-protein kinase Chk2	20
84	CHUK	Inhibitor of nuclear factor kappa-B kinase subunit alpha	20
85	ERBB3	Receptor tyrosine-protein kinase erbB-3	20
86	THBD	Thrombomodulin	18
87	NPM1	Nucleophosmin	18
88	ADRB2	Beta-2 adrenergic receptor	17
89	TOP1	DNA topoisomerase 1	17
90	BAX	Apoptosis regulator BAX	17
91	HMGCR	3-hydroxy-3-methylglutaryl-coenzyme A reductase	14
92	DUOX2	Dual oxidase 2	10
93	PIK3CG	Phosphatidylinositol 4,5-bisphosphate 3-kinase catalytic subunit gamma isoform	10
94	DRD2	D(2) dopamine receptor	9
95	ABCC1	Multidrug resistance-associated protein 1	9
96	LYZ	Lysozyme C	7
97	CHRM1	Muscarinic acetylcholine receptor M1	6
98	GSTM1	Glutathione S-transferase Mu 1	5
99	ALOX5AP	Arachidonate 5-lipoxygenase-activating protein	5
100	SLC22A5	Solute carrier family 22 member 5	5
101	SOAT1	Sterol O-acyltransferase 1	2

Abbreviations: QF Qingfei oral liquid; RSV respiratory syncytial virus.

**FIGURE 4:**
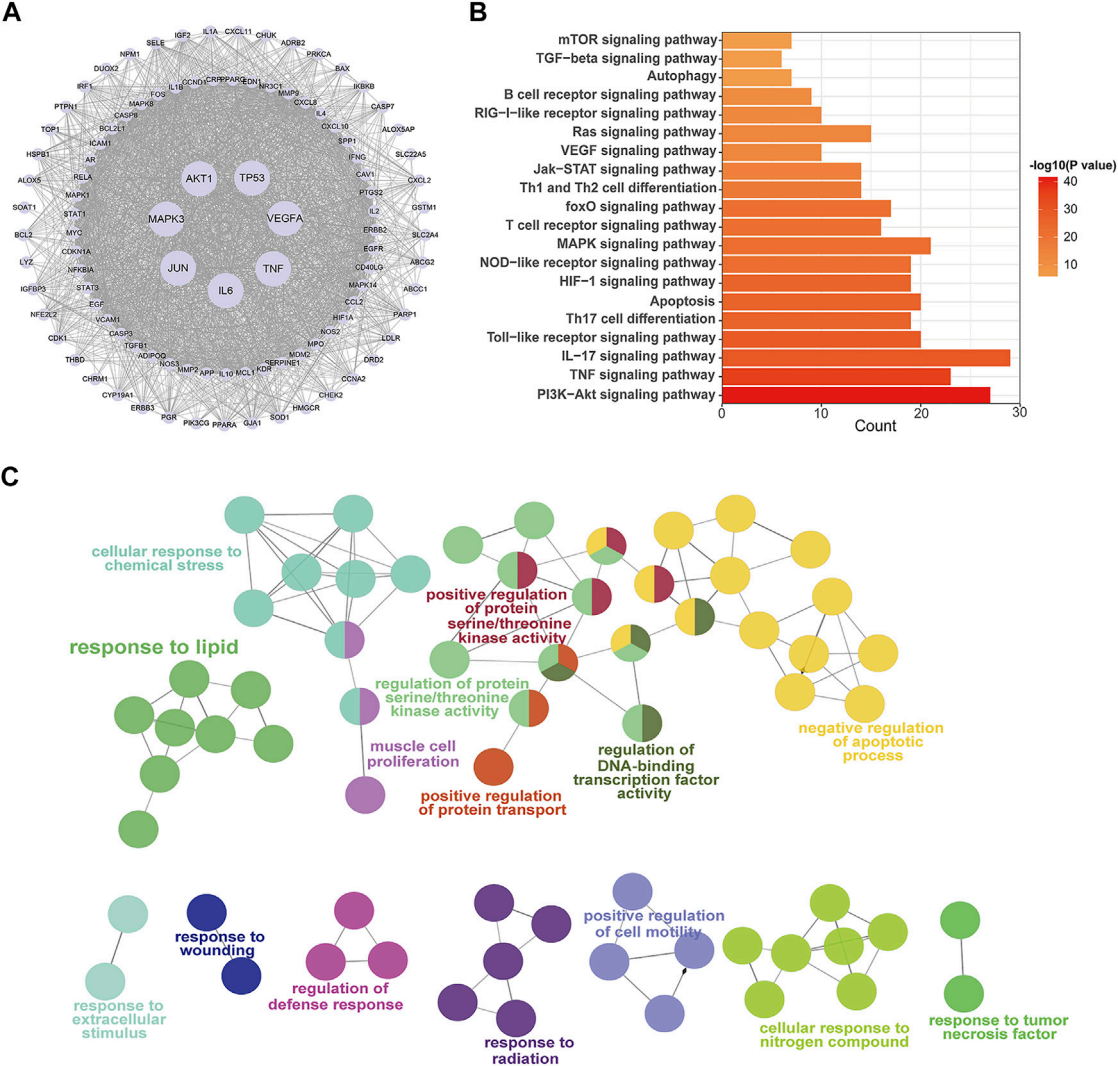
PPI network and enrichment pathway analysis for QF in the treatment of RSV-induced lung inflammation. **(A)** PPI network of core targets. Circle nodes represent core targets and edges represent interactions between them. The size of each node is positively correlated with the degree value. **(B)** KEGG pathway analysis of core targets. The top 20 pathways are ranked according to gene count and adjusted *p*-value (ap<0.05). **(C)** GO enrichment analysis of core targets. Circle nodes represent sub-branches of the main pathways. Nodes of the same color are clustered into one functional group. The name of each biofunction is shown next to the group with the same color. Edges represent interactions between sub-branches of one cluster. Abbreviations: QF, Qingfei oral liquid; RSV, respiratory syncytial virus; PPI, protein interaction.

The PPI network illustrated the relationship between core targets (Figure 4A). In the network, proteins including RAC-alpha serine/threonine-protein kinase (AKT1), Cellular tumor antigen p53 (TP53), Mitogen-activated protein kinase 3 (MAPK3), Vascular endothelial growth factor A (VEGFA), Tumor necrosis factor (TNF), Interleukin-6 (IL6), Transcription factor AP-1 (JUN), Caspase-3 (CASP3), Mitogen-activated protein kinase 8 (MAPK8), Signal transducer and activator of transcription 3 (STAT3), Prostaglandin G/H synthase 2 (PTGS2), Mitogen-activated protein kinase 1 (MAPK1), Pro-epidermal growth factor (EGF), Epidermal growth factor receptor (EGFR) and Myc proto-oncogene protein (MYC) were identified as hub nodes with a higher degree value.

The signaling pathways were shown using KEGG and GO analysis to better study the biofunctions of key targets involved in the QF against RSV-LI process. The top 20 KEGG pathways, as shown in Figure 4B, were mostly related to immunological function, inflammatory response, and cell stress. Because of the lowest ^a^ *p* value, the phosphatidylinositol 3-kinase (PI3K)/protein kinase B (AKT) signaling pathway was rated top. The downstream of PI3K/AKT signal transduction including mammalian target of rapamycin (mTOR) and forkhead box (FoxO) were also shown in the list. Furthermore, ClueGO software was used to cluster fifteen significant GO terms in order to visualize the biological process (BP), cellular component (CC), and molecular function (MF) of core targets. With enrichment ratios greater than 10%, the apoptotic process, protein serine/threonine kinase activity, lipid response, and cellar response to chemical stress were found to be the most significant (Figure 4C).

### Lipidomic Profiling of QF Against RSV-LI

**FIGURE 5:**
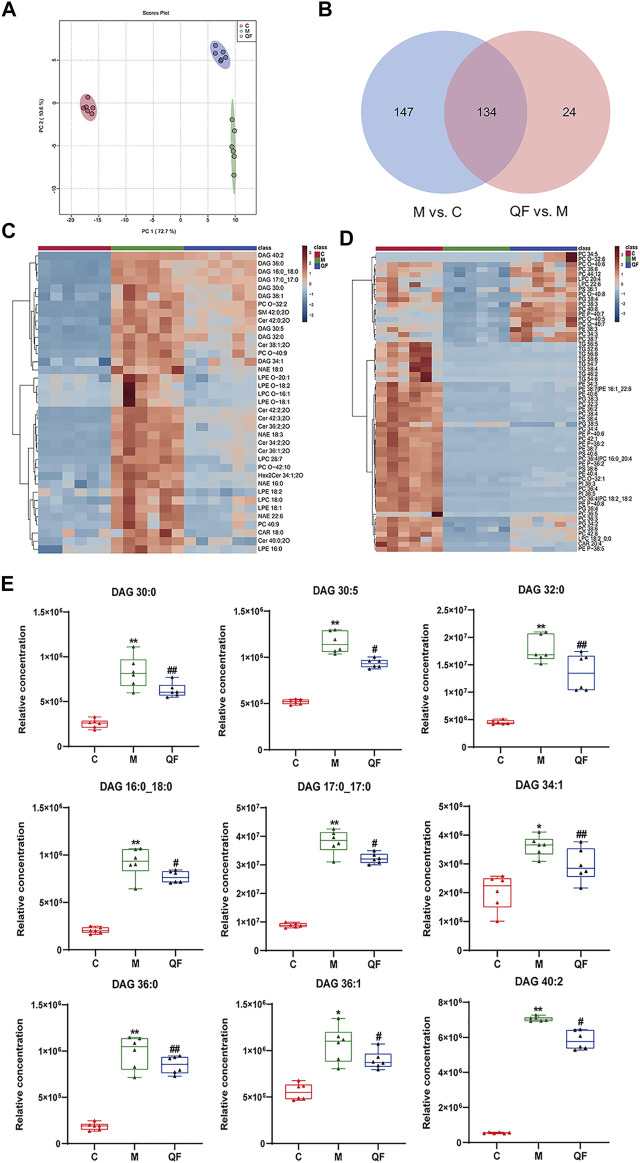
Effect of QF on lipid metabolism in lung tissues of mice with RSV-induced lung inflammation. **(A)** PCA score plot based on lipid profiles among control (C), model (M), and therapy (QF) groups. **(B)** Venn diagram of differential metabolites identified in M vs. C and QF vs. M comparisons. **(C)** Heatmaps of differential lipid metabolites up-regulated in M vs. C groups and down-regulated in QF vs. M groups. **(D)** Heatmaps of differential lipid metabolites down-regulated in M vs. C groups and up-regulated in QF vs. M group. **(E)** Boxplot of the relative concentrations of diglycerides (DAGs) in control (C), model (M), and therapy (QF) groups. * Compared with control group, # compared with model group, *p < 0.05, **p < 0.01, ^#^ *p* < 0.05, ^##^ *p* < 0.01). Abbreviations: QF, Qingfei oral liquid; RSV, respiratory syncytial virus; DAG diacylglycerol.

Untargeted lipidomics were applied based on our previous study that revealed decreased levels of lipids including triglyceride (TG) and glycerophosphates in RSV-infected mice (Shan et al., 2018), as well as the GO enrichment results of network pharmacology (Figure 3C) that connected to a lipid response. The principal component analysis (PCA) model in Figure 5A demonstrated full separations of lipidomic profiles across the control (C), model (M), and treatment (QF) groups. There were 281 differential lipids detected in the M vs. C group, 158 in the QF vs. M group, and 134 in both comparisons (Figure 5B). The heatmaps in Figures 5C,D were established using 97 lipids that were expressed differently in the M vs. C and QF vs. M groups. Diacylglycerol (DAG), N-acylethanolamine (NAE), and ceramide (Cer) were higher expressed in lung tissues of RSV-infected mice (versus control, *FDR*<0.05, FC>1.2), whereas triglyceride (TG) and glycerophospholipids including phosphatidylcholine (PC), phosphatidylethanolamine (PE), phosphatidylinositol (PI), phosphatidylglycerol (PG), phosphatidylserine (PS) were lower expressed. (versus control, *F*DR<0.05, FC<0.833). After QF therapy, all of these lipid metabolites recover to normal levels at various degrees.

In this investigation, however, there was no discernible change in the relative amounts of lysophospholipids and oxidized phospholipids (Figures 5C,D). Supplementary Table S3 provided detailed information of lipids in heatmaps. Above all, we found that QF controlled the lipidomic abnormalities produced by RSV infection in the lungs of mice, as evidenced by decreased DAG, Cer, NAE, and increased production of TG, PC, PE, PG, PS, and PI.

### QF Inhibited DAG Synthesis via PI3K/AKT/mTOR Pathway-Related Proteins

**FIGURE 6:**
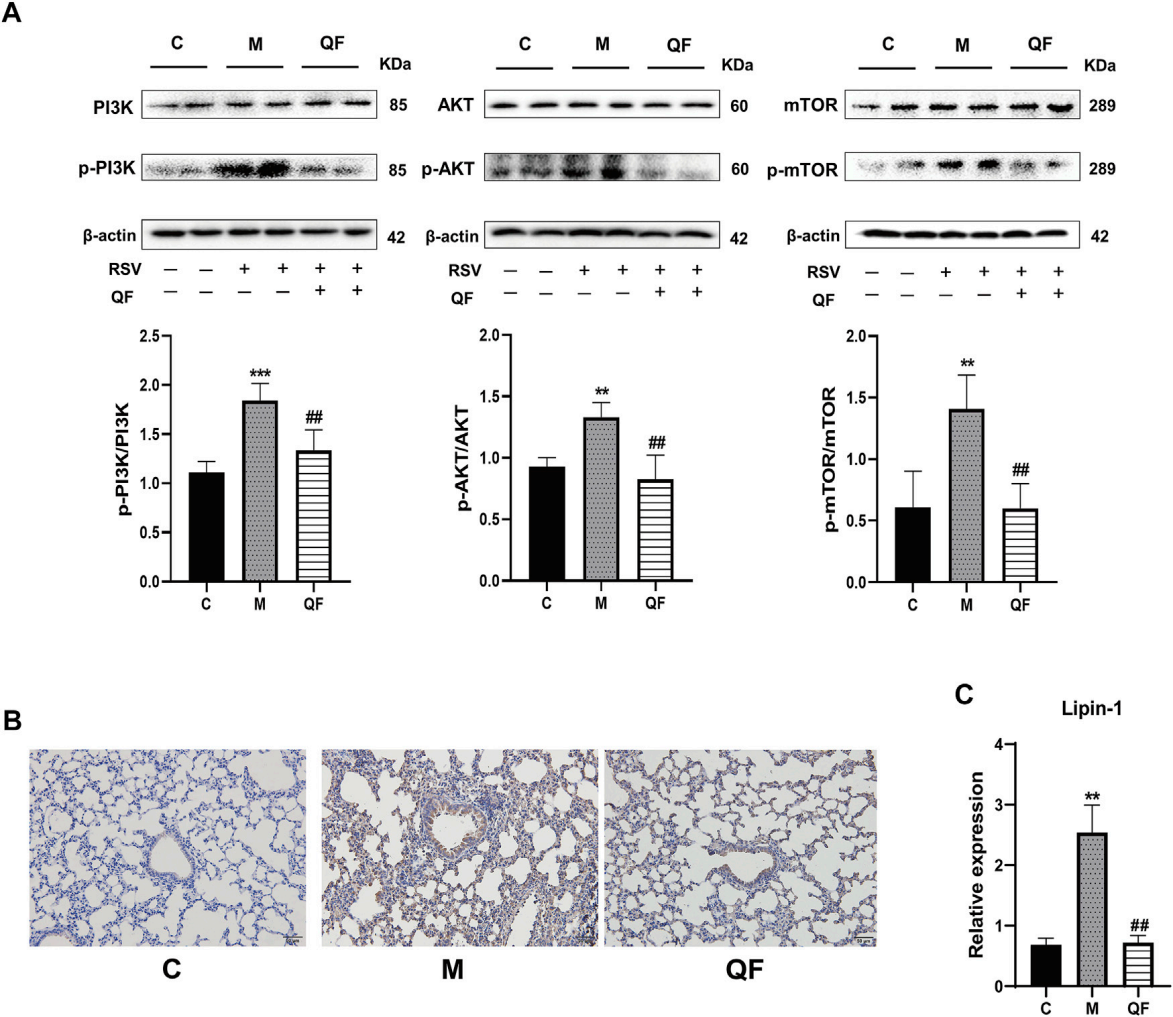
Effect of QF on the activity of the PI3K/AKT/mTOR/Lipin-1 signaling pathway in lung tissues of mice with RSV-induced lung inflammation. **(A)** Protein expression of phosphatidylinositol 3-kinase (PI3K), phospho-phosphatidylinositol 3-kinase (p-PI3K), protein kinase B (AKT), phospho-protein kinase B (p-AKT), mammalian target of rapamycin (mTOR), phospho-mammalian target of rapamycin (p-mTOR), and β-actin in control (C), model (M), and therapy (QF) groups. The densitometry value of each phosphorylated protein was normalized against its total abundance. Values are expressed as mean ± SEM (*n* = 4; * compared with control group, # compared with model group, **p < 0.01, ***p < 0.001, ^##^ *p* < 0.01). **(B)** Immunohistochemical staining of Lipin-1 protein in lung tissues of mice in control (C), model (M), and therapy (QF) groups (200 ×magnification). **(C)** mRNA expression level of lipin-1 protein in control (C), model (M), and therapy (QF) groups. Compared with control group, # compared with model group. **p < 0.01, ^##^ *p* < 0.01. Abbreviations: QF, Qingfei oral liquid; RSV, respiratory syncytial virus.

DAG was reported as the second messenger triggering signaling cascades in the previous study (Sim et al., 2020). We discovered that relative lung concentrations of DAG 30:0, DAG 30:5, DAG 32:0, DAG 16:0_18:0, DAG 17:0_17:0, DAG 34:1, DAG 36:0, DAG 36:1 and DAG 40:2 were elevated in model group, whereas QF suppressed lung levels of the DAGs in mice with RSV-induced LI (Figure 5E). As a result, we hypothesized that QF might have important therapeutic effects through modulating DAG synthesis. Lipin-1, a key enzyme in DAG production, was shown to be elevated in the lung tissue of mice with RSV-LI, and it was reduced following QF treatment (Figures 6B,C). In addition, lipin-1 serves as the downstream of the PI3K/AKT/mTOR signaling pathway (Peterson et al., 2011). The increased levels of phosphorylated versions of the proteins PI3K, AKT, and mTOR in the lung tissues of mice in the QF group were all reduced in our investigation (versus model, ^##^ *p*<0.01; Figure 6A). In RSV-LI mouse models, the data showed that QF could inhibit DAG synthesis via modulating the PI3K/AKT/mTOR signaling pathway and its related protein levels of lipin-1.

### QF Inhibited DAG-Induced Excessive Autophagy

**FIGURE 7:**
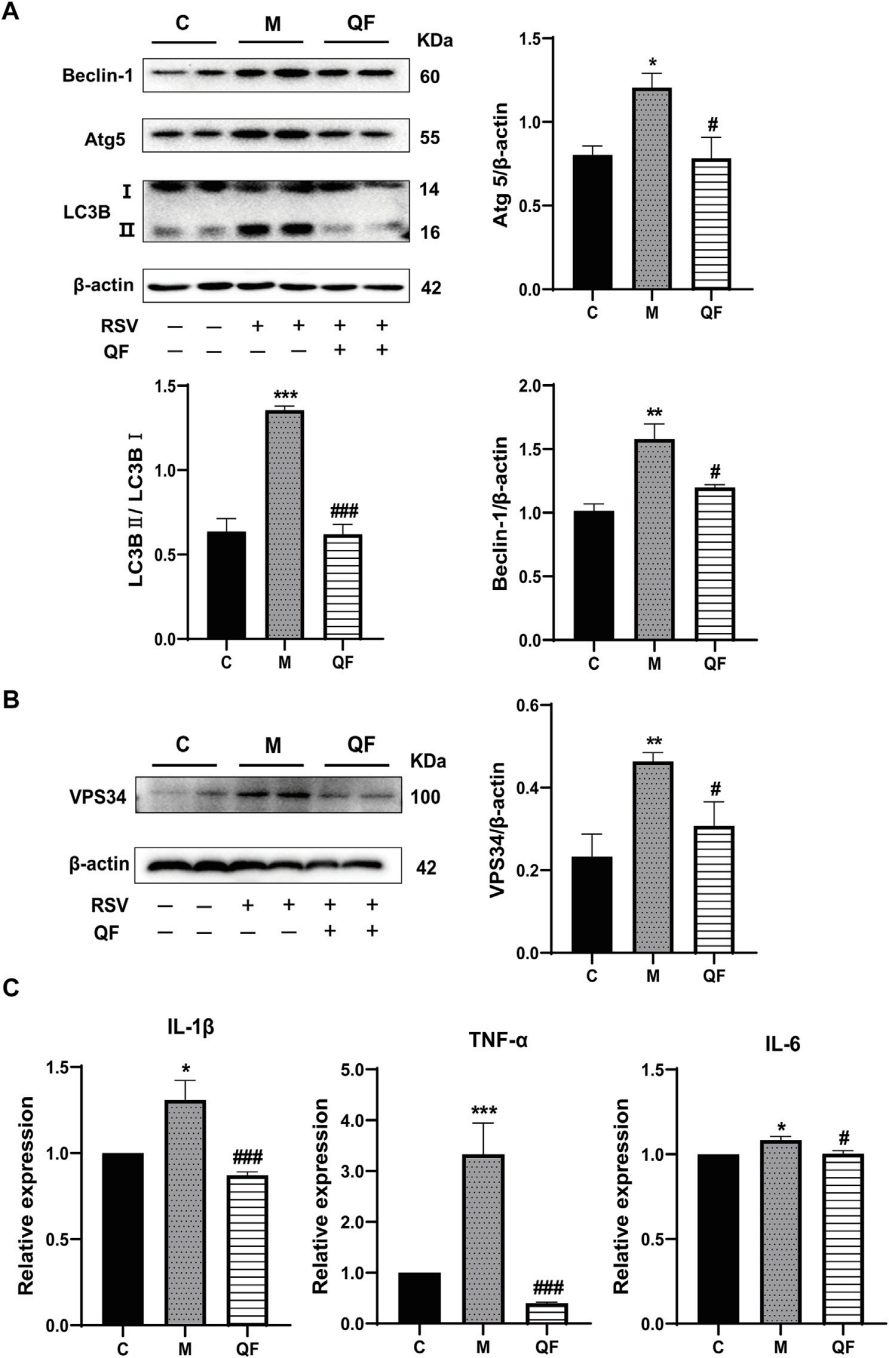
Effect of QF on DAG-induced autophagy and inflammation in lung tissues of mice with RSV-induced lung inflammation. **(A)** Protein expression of Beclin-1, Atg5, LC3B (I, II), and β-actin in control (C), model (M), and therapy (QF) groups. Densitometry values of Beclin-1 and Atg5 were normalized against β-actin to determine relative protein expression values. The densitometry value of LC3BII was normalized against LC3BI. Values are expressed as mean ± SEM (*n* = 3; * compared with control group, ^#^ compared with model group, *p < 0.05, **p < 0.01, ***p < 0.001, ^#^ *p* < 0.05, ^###^ *p* < 0.001). **(B)** VPS34 protein expression in control (C), model (M), and therapy (QF) groups. The densitometry value of VPS34 was normalized against β-actin. Values are expressed as mean ± SEM (n = 3; * compared with control group, ^#^ compared with model group, **p < 0.01, ^#^ *p* < 0.05). **(C)** Transcription levels of pro-inflammatory cytokines among control (C), model (M), and therapy (QF) groups. Values are expressed as mean ± SEM (*n* = 6; * compared with control group, # compared with model group, *p < 0.05, *** *p* < 0.001, ^#^ *p* < 0.05, ^###^ *p* < 0.001). Abbreviations: QF, Qingfei oral liquid; RSV, respiratory syncytial virus.

Previous study discovered that Lipin-1-mediated DAG synthesis might activate excessive autophagy via stimulating type III phosphatidylinositol kinase (VPS34) signaling (Zhang et al., 2014). In our study, VPS34 was triggered by RSV-LI and suppressed after QF treatment (versus control, *p<0.01; versus model, ^#^ *p*<0.05, Figure 7B). The top-ranked list of KEGG analysis in Figure 3B included autophagy, a highly conserved cellular recycling mechanism that is intimately connected to viral replication and immune response during RSV infection (Li et al., 2018; Oh et al., 2020). To see if such a pathogenic process was activated by RSV-LI, we detected the expression of autophagy-related proteins including Beclin-1, Atg5, and LC3B. Figure 7A showed that protein levels of Beclin-1, Atg5, and LC3B II were all increased in the model group (versus control, *p<0.05, **p<0.01, ***p<0.001), but significantly decreased in the QF group (versus model, ^#^ *p*<0.05, ^##^ *p*<0.01). Considering the close relationship between excessive autophagy and inflammation, our results demonstrated that QF effectively reduced high levels of proinflammatory cytokines, such as IL-1β, TNF-α and IL-6(versus control, *p<0.05, **p<0.01, ***p<0.001; versus model, ^#^ *p*<0.05, ^###^ *p*<0.001, Figures 7C–E). As a consequence, we conclude that QF can suppress DAG-induced excessive autophagy and inflammation in lung tissues of RSV-LI.

## Discussion

Applying an omics strategy in network research can improve the accuracy and efficiency of exploring the mechanisms underlying TCM treatment. Our study systematically clarified the underlying mechanism of QF in the treatment of RSV-LI using lipidomic-based network pharmacology and validated by animal experiments. With the help of bioinformatics and omics analyses, we revealed that QF alleviated RSV-LI by mediating DAG-related autophagy, which is regulated by the PI3K/AKT/mTOR signaling pathway and its related proteins lipin-1 and VPS34.

In the pharmacokinetic system, compounds in QF with OB ≥ 30% and DL ≥ 0.18 were considered active ingredients that could be absorbed and utilized in the human body. By constructing an active compound-core target network, we found that Quercetin was the most effective ingredient in QF for the treatment of RSV-LI, followed by Luteolin, Kaempferol, Naringenin, Tanshinone IIA, Beta-sitosterol, Cryptotanshinone, Ellagic acid, Isorhamnetin, and Dihydrotanshinlactone (Table 2). In terms of chemical properties, most ingredients were identified as flavonoids, natural polyphenols with presumed beneficial effects on anti-inflammation, antioxidation, and immunomodulation (Yi, 2018; Zeng et al., 2019). Previous findings confirmed that the main flavone constituents in QF, such as Quercetin and Luteolin, could inhibit RSV replication by directly blocking viral adhesion or modulating microRNA-related signaling pathways (Wang S. et al., 2020; Lopes et al., 2020). Although there was no literature reporting direct links between RSV and Kaempferol, Naringenin, or Isorhamnetin, these compounds are reported to play critical roles in antiviral and anti-inflammatory procedures by reducing viral replication, inhibiting inflammatory cytokines, and eliminating free radicals in respiratory tract viral diseases (Yang et al., 2019; Huang YF. et al., 2020). Beta-sitosterol, a common phytosterol in six herbs of QF, exerts its therapeutic effect by ameliorating virus-induced proinflammatory responses and modulating immunologic activity (Fraile et al., 2012; Zhou et al., 2020). Tanshinone IIA and Cryptotanshinone, two active ingredients found in Radix Salviae, exert antiviral activity by suppressing autophagy and apoptosis during viral proliferation (Sun et al., 2019; Huang C. et al., 2020). During fingerprinting analysis of QF, Quercetin, Luteolin, Kaempferol, Naringenin, and Tanshinone IIA were also identified by LTQ-Orbitrap MS through comparison with standards (Supplementary Figure S1). Consequently, the existing literature and our experimental results supported the network prediction, and demonstrated the successful application of a network pharmacology approach in identifying active compounds in TCM formulas.

From PPI network and pathway enrichment analyses, core targets including AKT1, TP53, MAPK3, VEGFA, TNF, IL6, JUN, and CASP3 were identified as potential major points of action for QF against RSV-LI, and these targets are closely associated with inflammation, apoptosis, and carcinogenesis in the progressive course of disease. Protein kinase B (AKT), the most significant target, is a critical serine/threonine kinase that mediates cellular survival, proliferation, migration, metabolism, and angiogenesis, and it also participates in signal transduction in the PI3K/AKT/mTOR pathway, which was enriched according to KEGG analysis (Figure 3B). A recent study claimed that AKT is a promising drug target for reducing tissue damage and helping to eliminate virus infection (Xia et al., 2020). Meanwhile, through RNA interference, several genes in the PI3K/AKT/mTOR signaling pathway were demonstrated to support broad-spectrum viral replication, and Everolimus, an mTOR inhibitor, showed apparent antiviral activity against many kinds of viruses in vitro experiments (Murray et al., 2012). To further investigate the effect of QF on this signaling pathway during RSV infection, we measured the expression of crucial proteins by using western blotting. The results demonstrated the activation of this signaling pathway in lung tissues of mice in the treatment group, whereas QF exerted an inhibitory effect during treatment (Figure 6A).

The PI3K/AKT/mTOR signaling pathway involves upstream effectors that regulate a broad range of cellular processes including survival, proliferation, and growth (Ersahin et al., 2015). In primary metabolism, the PI3K/AKT/mTOR axis promotes various diseases by activating lipid synthesis (Chen et al., 2019; Yi et al., 2020). During viral infection, host invasion requires lipid modification for cell entry and trafficking, and the reprogramming of lipid metabolism and compartmentalization for assembly and egress (Mazzon and Mercer, 2014). In the present study, we also found that the lipid response was involved in the process by which QF treats RSV-LI, according to GO enrichment (Figure 4C). Through lipidomic analysis, 97 lipids were identified as metabolites that conversely expressed between M vs. C and QF vs. M groups. Among them, the abundance of DAG, NAE, and Cer in lung tissues was decreased after QF treatment, while the opposite was true for TG, PC, PE, PI, PS, and PG (Figures 5C,D). Moreover, boxplots of DAG 30:0, DAG 30:5, DAG 32:0, DAG 16:0_18:0, DAG 17:0_17:0, DAG 34:1, DAG 36:0, DAG 36:1, and DAG 40:2 among the three groups further confirmed that DAG synthesis was accelerated in the RSV-infected group but suppressed in the treatment group (Figure 5E). Regarding the link between the PI3K/AKT/mTOR pathway and DGA synthesis, lipin-1 is a key phosphatidic acid phosphatase (PAP) enzyme and the main factor in DAG production. In agreement with the mTOR signaling changes, the abundance of Lipin-1 protein was observed to be increased in mice with RSV-LI and decreased following QF therapy in immunohistochemical investigations, and this tendency was validated by PCR in mRNA levels (Figures 6B,C). These observations indicate that the PI3K/AKT/mTOR pathway may stimulate the synthesis of DAG by acting on the lipin-1 target.

Mammalian lipin proteins are involved in many cellular processes such as lipid storage, lipoprotein synthesis, autophagy, and gene expression (Reue and Wang, 2019). Previous studies on autophagy reported that QF could inhibit this process induced by RSV infection via the mTOR signaling pathway, but the specific regulatory mechanism has not been clarified (Yu et al., 2021). As a key enzyme mediated by mTOR signaling, the initiation of mature autophagosomes requires lipin-1 phosphatidic phosphatase activity to generate DAG at the surface of autophagosomes/lysosomes, which further activates the VPS34 signaling cascade to form functional autolysosomes (Zhang et al., 2014). This mechanism was verified via *in vivo* experiments showing that RSV-induced autophagy was accompanied by the activation of lipin-1 protein, DAG generation, and subsequent autophagy-related VPS34 signaling, and all were inhibited by QF treatment (Figures 7A,B).

**FIGURE 8:**
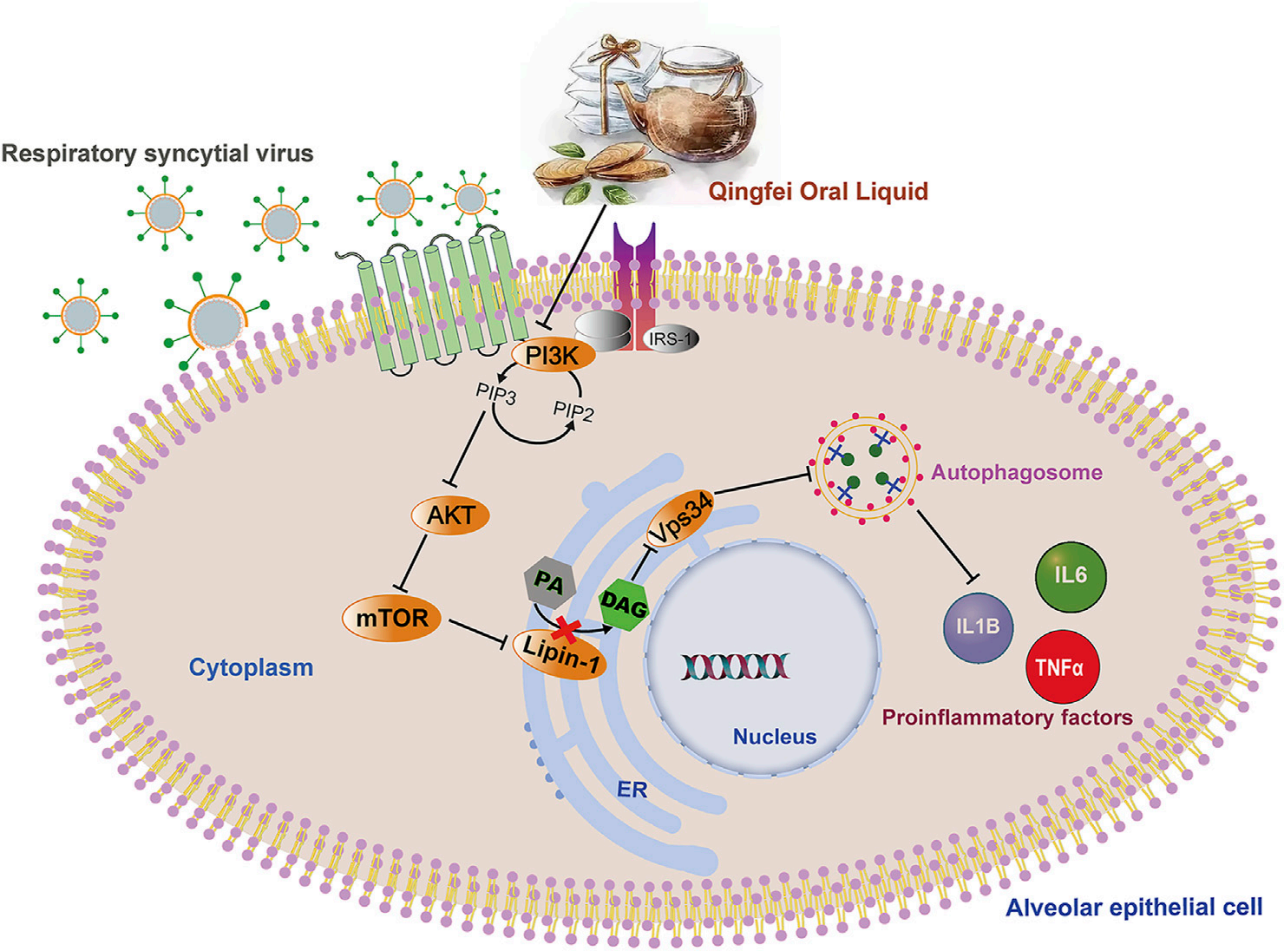
Overall regulation mediated by QF in the treatment of RSV pneumonia. Abbreviations: QF, Qingfei oral liquid; RSV, respiratory syncytial virus.

Autophagy is a common intracellular self-digestion process in the human body that delivers intracellular cargo to lysosomes to generate autophagosomes, in which the contents are degraded or recycled (Painter et al., 2020). As protective or maladaptive responses, the roles of autophagy in the development of lung diseases remain poorly understood (Racanelli et al., 2018). Crosstalk between autophagy and the immune system helps maintain homeostasis and physiological functions to protect against infectious, autoimmune, and inflammatory diseases (Levine et al., 2011). However, once the balance is broken, excessive autophagy may lead to autophagic death of immune cells, which further aggravates the inflammatory response (Qiu et al., 2019). Recent findings proved that persistent or inefficient autophagy was detrimental to lung epithelial cells and promoted lung injury (Zhao et al., 2019). Consistently, both H&E staining and transcription measurements in the present work supported the inflammatory reaction in lung tissues in RSV-infected groups (Figures 2D, 7C), and this pathological progression may be closely related to dysregulated autophagy induced by RSV infection. Taken together, our findings suggest that QF may inhibit autophagy mainly via the regulation of DAG synthesis through the PI3K/AKT/mTOR signaling pathway and its related proteins lipin-1 and VPS34 (Figure 8). Detailed exploration of the underlying mechanism of QF against RSV-LI will be conducted in future studies.

## Conclusion

In conclusion, this study investigated the pharmacological mechanism of QF in treating RSV-LI with the combination of network pharmacology, lipidomics and experimental validation. According to the observations, we suggested that QF may inhibit RSV-induced DAG synthesis via suppressing PI3K/Akt/mTOR signaling pathway and its related proteins lipin-1 and VPS34, and this process will further reduce the excessive autophagy combined with associated inflammation. Therefore, the roles of DAG synthesis and autophagy may provide potential avenues for the therapeutic targeting of RSV-LI.

## Data Availability

The original contributions presented in the study are included in the article/Supplementary Material, further inquiries can be directed to the corresponding authors.
