# Supplementary material for: Integrated Network Pharmacology and Lipidomics to Reveal the Inhibitory Effect of Qingfei Oral Liquid on Excessive Autophagy in RSV-Induced Lung Inflammation
Source: Front Pharmacol. 2021 Dec 1;12:777689. doi: 10.3389/fphar.2021.777689 (PMC8672039; doi:10.3389/fphar.2021.777689)

Autophagy

Atg5

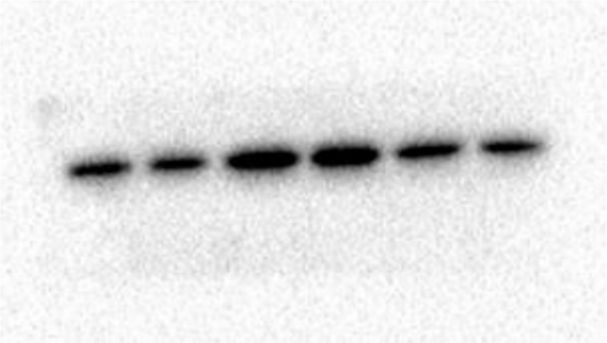

Beclin-1

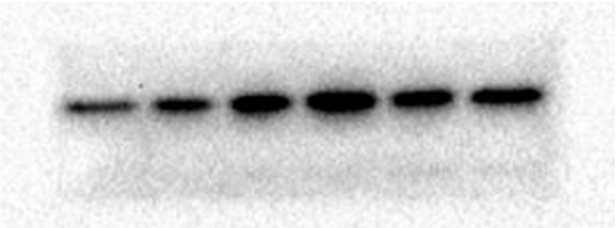

LC3B

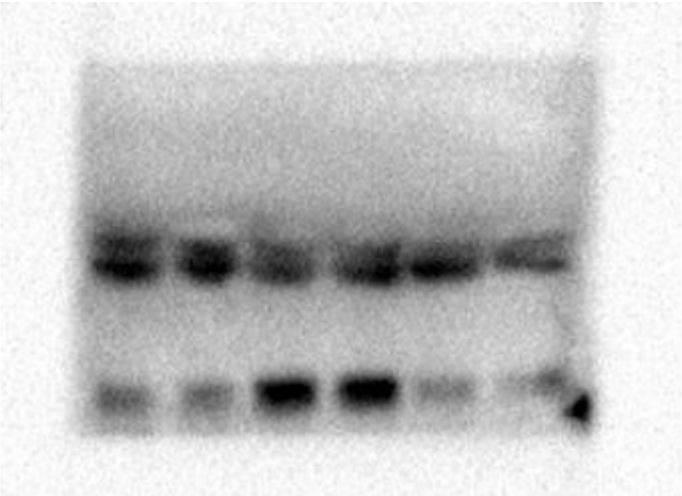

β-actin (Atg5/Beclin-1/LC3B)

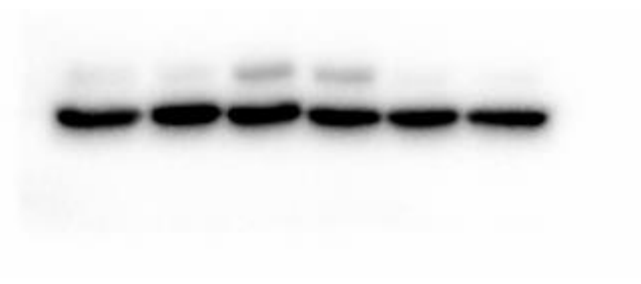

VPS 34

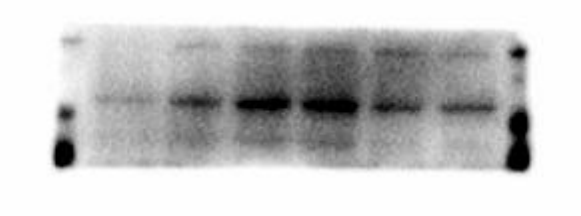

β-actin (VPS 34)

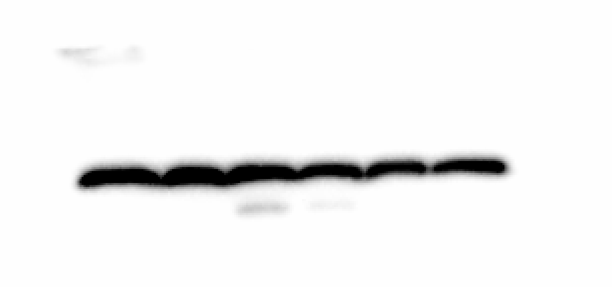

PI3K/AKT/mTOR signaling

PI3K

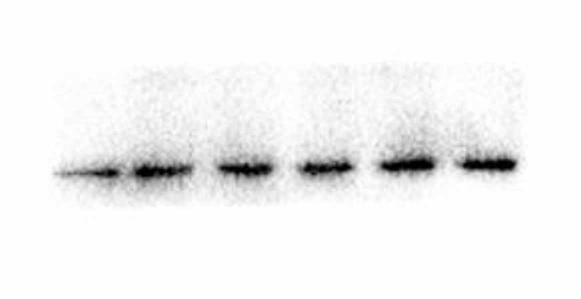

p-PI3K

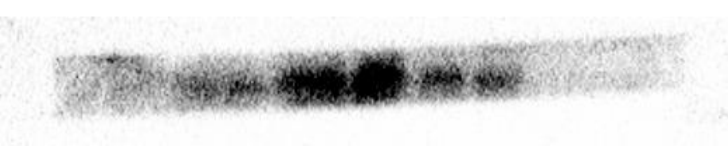

β-actin (PI3K)

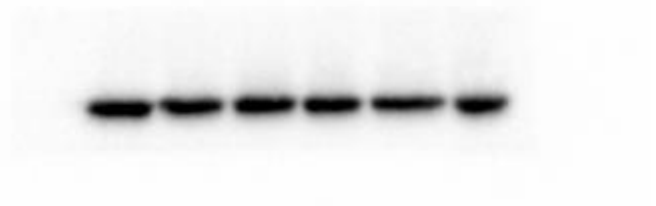

AKT

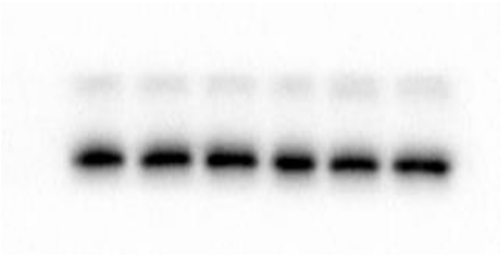

p-AKT

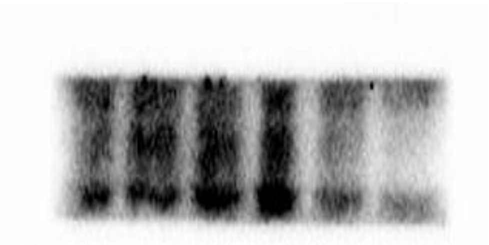

β-actin (AKT)

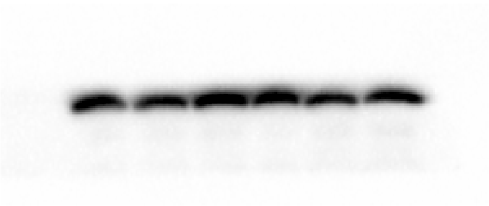

mTOR

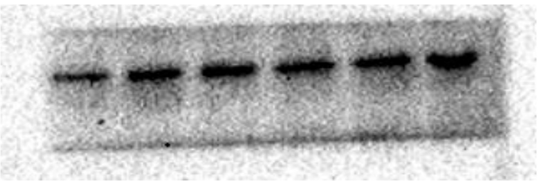

p-mTOR

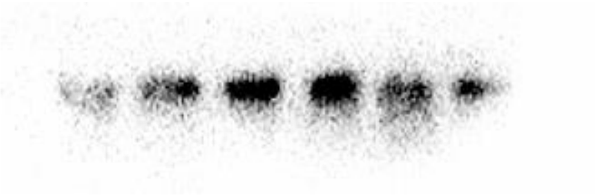

β-actin (mTOR)

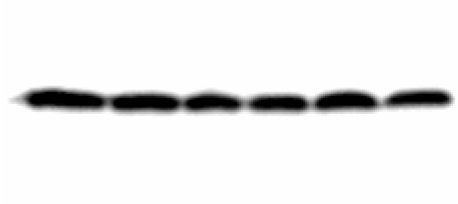

Immunohistochemistry

Control

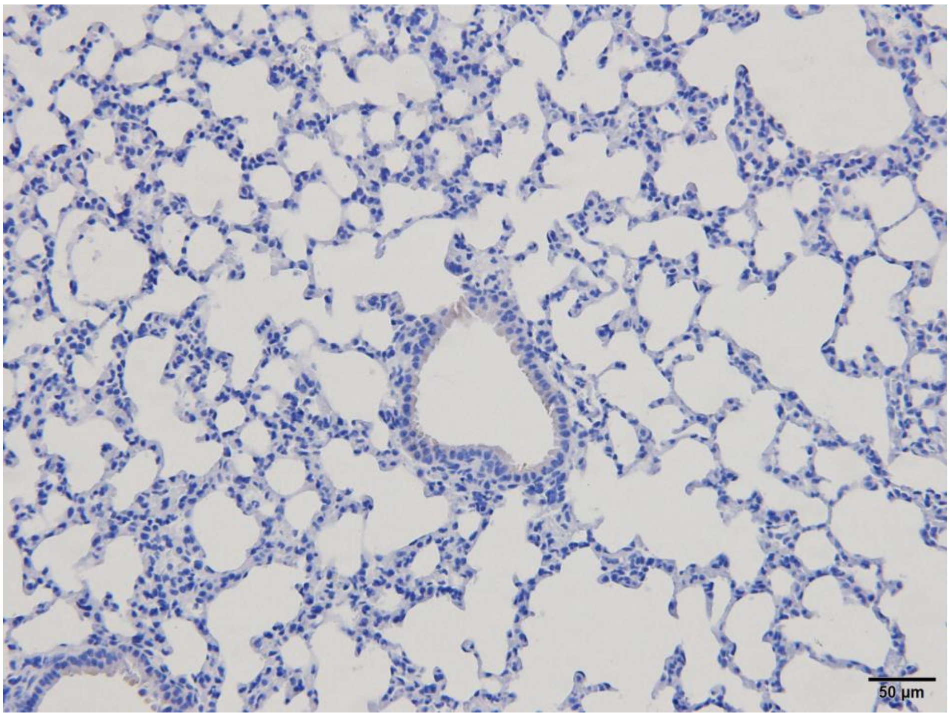

Model

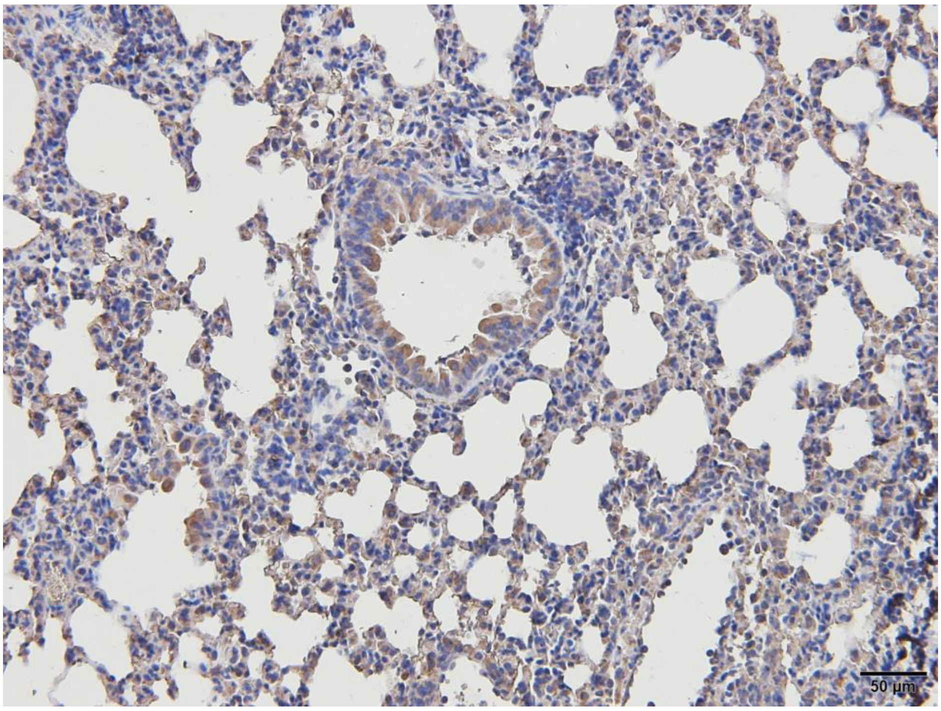

QF

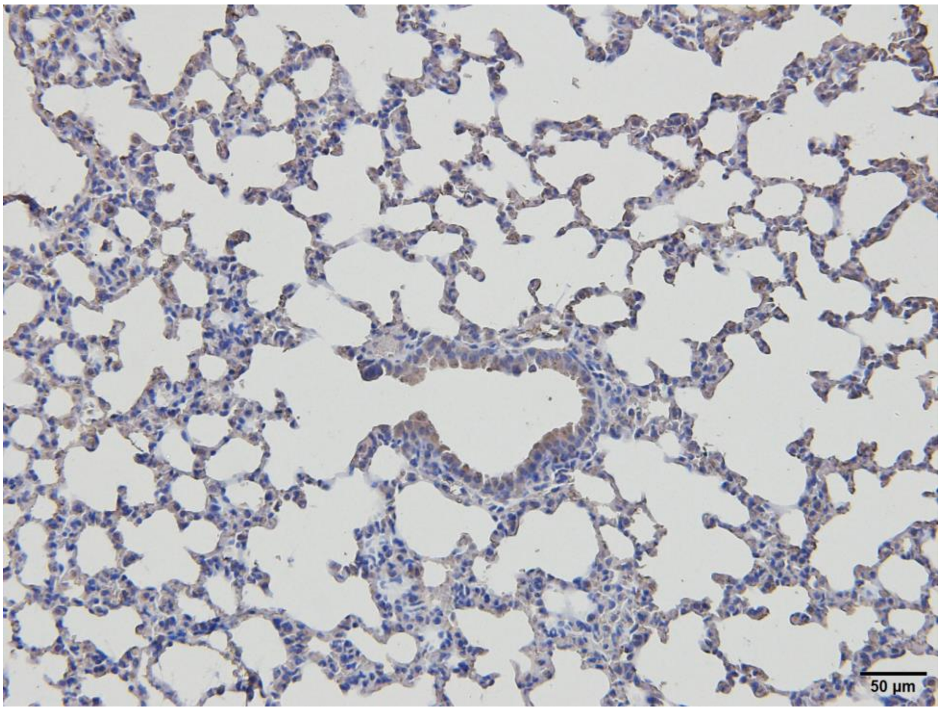

Supplement: Supplementary file 1 [file DataSheet6.pdf]
