# Supplementary material for: Integrated Network Pharmacology and Lipidomics to Reveal the Inhibitory Effect of Qingfei Oral Liquid on Excessive Autophagy in RSV-Induced Lung Inflammation
Source: Front Pharmacol. 2021 Dec 1;12:777689. doi: 10.3389/fphar.2021.777689 (PMC8672039; doi:10.3389/fphar.2021.777689)
Supplement: Supplementary file 3 [file DataSheet1.doc]

**Integrated Network Pharmacology and Lipidomics to Reveal the Inhibitory effect of Qingfei Oral Liquid on excessive autophagy in RSV-induced Lung Inflammation**

**Running title: Lipidomics-based Network Pharmacology of QF**

Li An^1†^, Lili Lin^1†*^, Hui Chen^1^, Lu Feng^1^, Tong Xie^1^, Cunsi Shen^1^, Jianjian Ji^1^, Mengjiang Lu^2^, Yuling Liu^3^, Jinjun Shan^1^, Xiaorong Wang^4^*, Shouchuan Wang^1*^

^1^Jiangsu Key Laboratory of Pediatric Respiratory Disease, Institute of Pediatrics, Medical Metabolomics Center, Affiliated Hospital of Nanjing University of Chinese Medicine, Nanjing, China, ^2^Key Laboratory of Acupuncture and Medicine Research of Ministry of Education, Nanjing University of Chinese Medicine, Nanjing, China, ^3^Department of Pediatrics, Nanjing Pukou District Hospital of Traditional Chinese Medicine, Nanjing, China, ^4^Department of Clinical Laboratory, Affiliated Hospital of Nanjing University of Chinese Medicine, Nanjing, China

**Supporting methods of the quality control of QF**

Take 100μL of Qingfei oral liquid and add methanol 800μL, put the diluted solution in a centrifuge at 4 ◦C and 18, 000 rpm for 10 min, take supernatant and inject sample for analysis. LC-MS/MS analysis of the supernatant was performed on Linear Ion Trap Quadrupole-Orbitrap Mass Spectrometer (LTQ-Obitrap MS; Thermo Fisher Scientific, USA) under positive and negative ionization modes. LC separation was run on Acquity UPLC HSS T3 analytical column (2.1 × 100 mm, 1.8 μm, Waters) with an ACQUITY UPLC H-CLASS system. Gradient elution was used with Acetonitrile and 0.1% formic acid in water as solvent A and 0.1% formic acid in water as solvent B, with a flow rate of 0.2 ml/min. Initial conditions were 10% solvent B, gradually increased to 90% B at 26 min, then decreased to 10% B at 28min, and 2 min of re-equilibration time was used to ensure retention time stability. Samples were kept at 4◦C and the injection volume was 3 μL. MS parameters were as follows: Nitrogen was utilized as the sheath gas and auxiliary gas, with flow rates set at 45 and 15 arbitrary units, respectively. The scan ranges were100–1000m/z. The spray and capillary voltages were set between 3.5V and 4kV, respectively, the source temperature was set to 300 °C, and the tube lens was set to 110V in positive and negative ion mode.


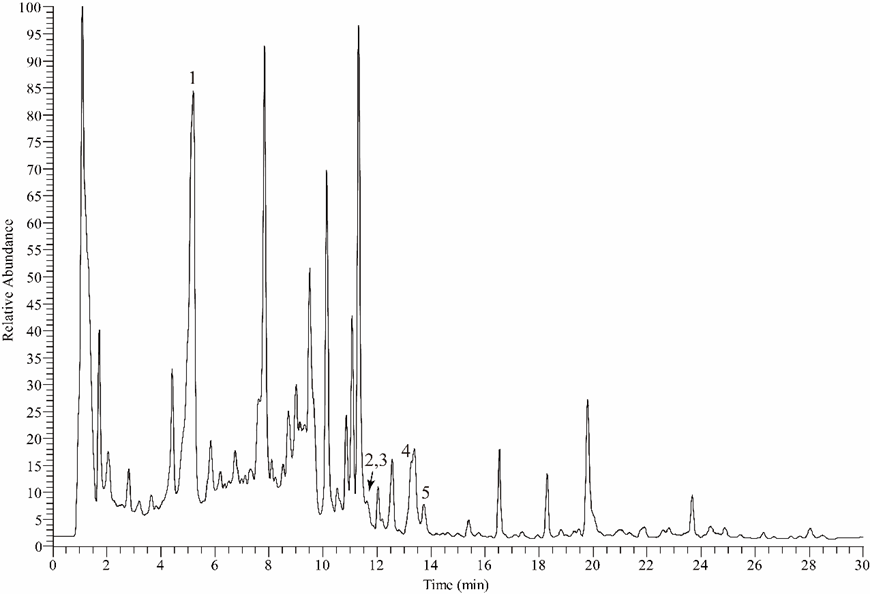


**B**


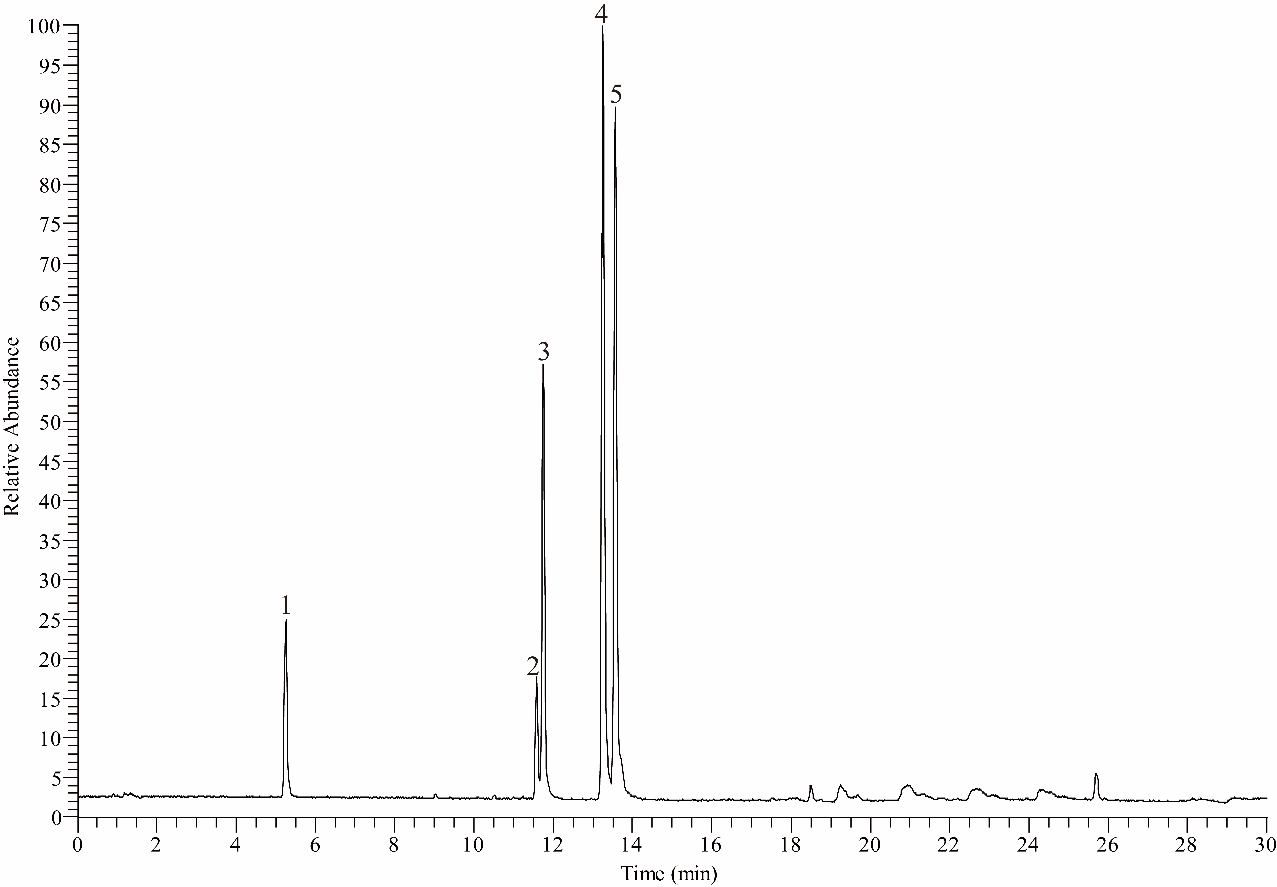


**A**


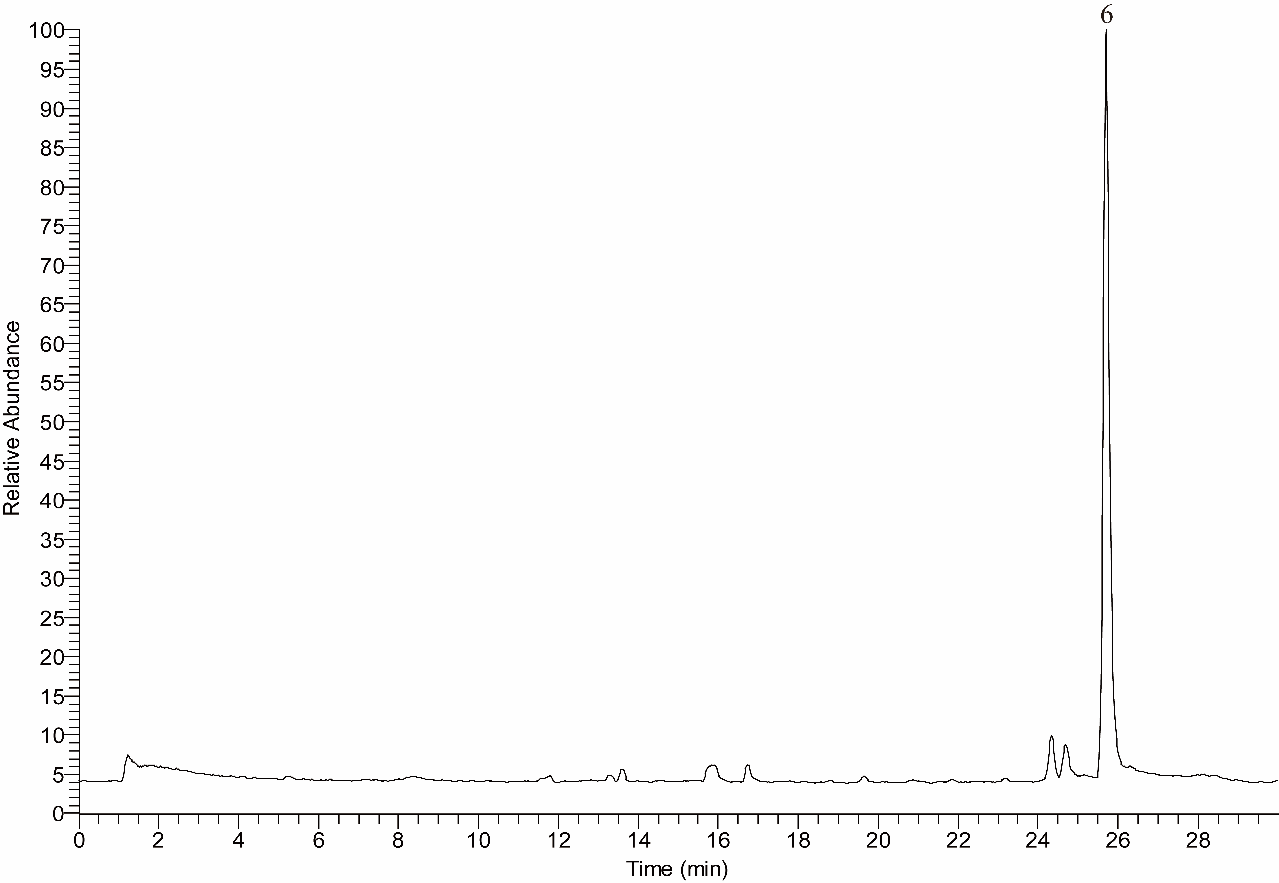


**C**


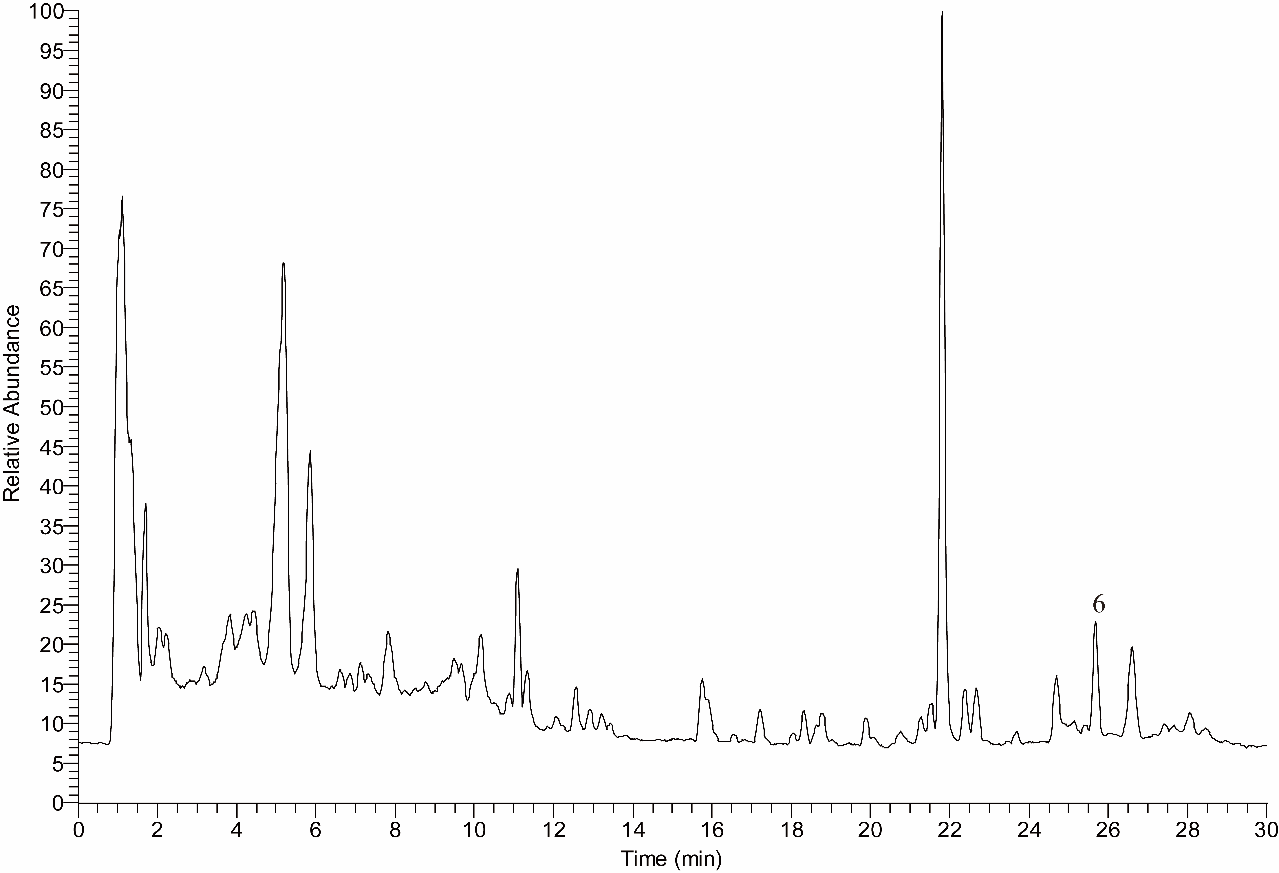


**D**

**Figure S1 Total ion chromatograms of QF and standard compounds in both positive and negative ionization modes.** (A) Representative chromatograms of standard compounds in the positive ionization mode. (B) Representative chromatograms of QF in the positive ionization mode. (C) Representative chromatograms of standard compounds in the negative ionization mode. (D) Representative chromatograms of QF in the negative ionization mode. (1) (+)-Catechin; (2) Luteolin; (3) Quercetin; (4) Naringenin; (5) Kaempferol; (6) Tanshinone IIA. Abbreviations: QF Qingfei oral liquid.

**Figure S1 Total ion chromatograms of QF and standard compounds in both positive and negative ionization modes.** (A) Representative chromatograms of standard compounds in the positive ionization mode. (B) Representative chromatograms of QF in the positive ionization mode. (C) Representative chromatograms of standard compounds in the negative ionization mode. (D) Representative chromatograms of QF in the negative ionization mode. (1) Catechin; (2) Luteolin; (3) Quercetin; (4) Naringenin; (5) Kaempferol; (6) Tanshinone IIA. Abbreviations: QF Qingfei oral liquid.
